# Psychological, social, and welfare interventions for torture survivors: A systematic review and meta-analysis of randomised controlled trials

**DOI:** 10.1371/journal.pmed.1002919

**Published:** 2019-09-24

**Authors:** Aseel Hamid, Nimisha Patel, Amanda C. de C. Williams

**Affiliations:** 1 Research Department of Clinical, Educational and Health Psychology, University College London, London, United Kingdom; 2 Clinical Psychology, School of Psychology, University of East London, London, United Kingdom; 3 International Centre for Health & Human Rights, London, United Kingdom; Addis Ababa University / King's College London, ETHIOPIA

## Abstract

**Background:**

Torture and other forms of ill treatment have been reported in at least 141 countries, exposing a global crisis. Survivors face multiple physical, psychological, and social difficulties. Psychological consequences for survivors are varied, and evidence on treatment is mixed. We conducted a systematic review and meta-analysis to estimate the benefits and harms of psychological, social, and welfare interventions for torture survivors.

**Methods and findings:**

We updated a 2014 review with published randomised controlled trials (RCTs) for adult survivors of torture comparing any psychological, social, or welfare intervention against treatment as usual or active control from 1 January 2014 through 22 June 2019. Primary outcome was post-traumatic stress disorder (PTSD) symptoms or caseness, and secondary outcomes were depression symptoms, functioning, quality of life, and adverse effects, after treatment and at follow-up of at least 3 months. Standardised mean differences (SMDs) and odds ratios were estimated using meta-analysis with random effects. The Cochrane tool was used to derive risk of bias. Fifteen RCTs were included, with data from 1,373 participants (589 females and 784 males) in 10 countries (7 trials in Europe, 5 in Asia, and 3 in Africa). No trials of social or welfare interventions were found. Compared to mostly inactive (waiting list) controls, psychological interventions reduced PTSD symptoms by the end of treatment (SMD −0.31, 95% confidence interval [CI] −0.52 to −0.09, *p* = 0.005), but PTSD symptoms at follow-up were not significantly reduced (SMD −0.34, 95% CI −0.74 to 0.06, *p* = 0.09). No significant improvement was found for PTSD caseness at the end of treatment, and there was possible worsening at follow-up from one study (*n* = 28). Interventions showed no benefits for depression symptoms at end of treatment (SMD −0.23, 95% CI −0.50 to 0.03, *p* = 0.09) or follow-up (SMD −0.23, 95% CI −0.70 to 0.24, *p* = 0.34). A significant improvement in functioning for psychological interventions compared to control was found at end of treatment (SMD −0.38, 95% CI −0.58 to −0.18, *p* = 0.0002) but not at follow-up from only one study. No significant improvement emerged for quality of life at end of treatment (SMD 0.38, 95% CI −0.28 to 1.05, *p* = 0.26) with no data available at follow-up. The main study limitations were the difficulty in this field of being certain of capturing all eligible studies, the lack of modelling of maintenance of treatment gains, and the low precision of most SMDs making findings liable to change with the addition of further studies as they are published.

**Conclusions:**

Our findings show evidence that psychological interventions improve PTSD symptoms and functioning at the end of treatment, but it is unknown whether this is maintained at follow-up, with a possible worsening of PTSD caseness at follow-up from one study. Further interventions in this population should address broader psychological needs beyond PTSD while taking into account the effect of multiple daily stressors. Additional studies, including social and welfare interventions, will improve precision of estimates of effect, particularly over the longer term.

## Abstract 2

Aseel Hamid and colleagues show that psychological interventions help alleviate PTSD symptoms in the short term for torture victims; long-term effects are unknown.

## Author summary

**Why was this study done?:**

- Torture occurs in the majority of countries around the world, often leaving survivors with prolonged physical and psychological problems. We still do not know what treatment for psychological problems is effective.
- This review aimed to calculate the effects of psychological, social, and welfare interventions on the mental health, functioning, and quality of life of torture survivors.

**What did the researchers do and find?:**

- Published data from 15 randomised controlled trials (RCTs)—all of psychological interventions, including 1,373 participants across 10 countries—were systematically reviewed and analysed.
- Compared to control conditions, psychological interventions significantly reduced symptoms of post-traumatic stress disorder (PTSD) and improved functioning at the end of treatment, but not at follow-up.
- Psychological interventions did not significantly improve depression symptoms or quality of life.
- Psychological interventions did not significantly reduce the incidence of PTSD diagnosis, and one study, with 28 participants, showed an increase of PTSD diagnosis at follow-up compared to control conditions.

**What do these findings mean?:**

- More effective interventions are needed with this population.
- Future interventions for torture survivors should be more collaborative and should address broader psychological problems beyond PTSD as well as social and welfare needs that maintain psychological problems.

## Introduction

Despite 156 countries having signed the United Nations Convention Against Torture and Other Cruel, Inhuman or Degrading Treatment and Punishment [1], torture is widespread, and Amnesty International has documented torture and other forms of ill treatment in 141 countries in 2014 [2]. Long-standing and ongoing armed conflict has likely led to the increased use of torture since. Worldwide, 352,000 fatalities resulting from organised violence were identified between 2014 and 2016 alone [3]. The prevalence of torture and resulting fatalities are likely higher but difficult to estimate given that perpetrators often obscure the use of torture, and there are multiple barriers to disclosure for survivors.

Torture has psychological, physical, social, and spiritual impacts that interact in diverse ways. Psychological effects are well documented; predominantly post-traumatic stress, depression, anxiety, and phobias [4,5]. Physical effects are also diverse (for reviews, see [6,7]). In addition, torture survivors’ disrupted lives can bring social and financial problems that contribute to and maintain psychological distress, whether as a refugee or in the country of origin [5,8,9].

Torture often occurs against a backdrop of national and international power imbalances, war, civil unrest, and the destruction or erosion of medical and other welfare services. Arguably, treatment needs to incorporate wider conceptualisations of damage and distress than are represented in standard Western psychological treatments for psychological trauma [10,11]. A review conducted in 2011 describes a limited range of interventions for torture survivors, tested in studies with significant limitations such as small sample sizes and unvalidated outcomes [6]. Given the scant literature, greater understanding of what works in treatment and rehabilitation for torture survivors is crucial in order to obtain maximum benefits from scarce resources.

A Cochrane systematic review and meta-analysis [12] aimed to summarise psychological, social, and welfare interventions for torture survivors but found eligible studies only of psychological treatment. The 9 randomised controlled trials (RCTs) included provided data on 507 adults with no immediate benefits for psychological therapy for psychological distress (as measured by depression symptoms), post-traumatic stress disorder (PTSD) symptoms, PTSD caseness, or quality of life. At follow-up, 4 studies with 86 participants showed moderate effect sizes in reducing psychological distress and PTSD symptoms. Conclusions were tentative, given the low quality of evidence, with underpowered studies and outcomes assessed in nonstandard ways, and no study assessed participation in community life or social and family relationships.

More recently, a meta-analysis of 18 pre-post studies of interventions for survivors of mass violence in low- and middle-income countries showed a large improvement in PTSD and depression across treatment [13] but smaller effects from controlled studies. Another recent review [14] concluded that cognitive behavioural therapy (CBT) interventions produced the best treatment outcome for PTSD and/or depression. However, both reviews recruited more widely than torture survivors. No recent systematic reviews or meta-analyses have focused on interventions for torture survivors. We conducted this systematic review and meta-analysis to assess the reported benefits or adverse outcomes in the domains of PTSD symptoms, PTSD caseness, psychological distress, functioning, and quality of life for psychological, social, and welfare interventions for torture survivors.

## Methods

### Search strategy and selection criteria

A systematic review was performed using the Preferred Reporting Items for Systematic Reviews and Meta-Analyses (PRISMA) statement [15], which is available in S1 PRISMA Checklist. To be included, studies had to be RCTs or quasi-RCTs of psychological, social, or welfare interventions for survivors of torture against any active or inactive comparison condition; the same criteria were used as in the previous review [12], and the full protocol is provided in S1 Text. Quasi-RCTs, in which the method of allocation is known but not strictly random—such as the use of alternation, date of birth, and medical record number [16]—were included considering the difficulties of conducting RCTs in this population.

We extracted RCTs from searches of PsycINFO, MEDLINE, EMBASE, Web of Science, the Cumulative Index to Nursing and Allied Health Literature, Cochrane Central Register of Controlled Trials, the WHO International Clinical Trials Registry Platform, Clinical Trials.Gov, PTSDpubs, and the online library of Danish Institute Against Torture (DIGNITY) databases from 1 January 2014 (1 January 2013 in the case of Web of Science, the Cumulative Index to Nursing and Allied Health Literature, and PTSDpubs) through 22 June 2019 using key search terms including combinations of “torture,” “randomised,” “trial,” and “intervention” with Boolean operators (S1 Text). There was no language restriction. We also searched reference lists of torture-specific reviews published in or after January 2014 and those emerging from the final set of included studies. We contacted corresponding authors when full texts were unavailable.

### Data extraction

We initially screened titles and abstracts against the inclusion criteria, with the aim of identifying potentially eligible studies for which the full paper was obtained. One author (AH) initially screened titles and abstracts to select full papers; another author (AW) checked a subsample of the excluded papers and agreed with all exclusions. Full papers were screened and selected for inclusion by 2 authors independently and agreed upon after discussion (AH and AW).

Descriptive data, including participant characteristics, treatment mode, and setting, were collected. The primary area of interest for this review was outcomes in the domains of PTSD symptoms and caseness, psychological distress, functioning, and quality of life. PTSD symptoms were defined as the primary outcome given that the majority of identified reviews measured this. Psychological distress was measured as a secondary outcome, in the form of depression symptoms. Depression was chosen to define psychological distress because it is more distinct from PTSD than alternative scaled constructs of psychological distress, particularly anxiety. As in Patel and colleagues’ review [12], functioning was measured by engagement in education, training, work, or community activity, and quality of life was defined as a change (positive or negative) in quality of life or well-being as measured by global satisfaction with life and extent of disability.

### Statistical analyses

Studies in which a psychological, social, or welfare intervention was an active treatment of primary interest were investigated. When studies included more than one arm within a trial, it was decided that—where both arms represented the same content of intervention—data from those arms were combined. The respective control arms associated with these intervention arms were also combined, given that the main area of interest of this research is the impact of intervention relative to control. In studies in which both adjusted and unadjusted treatment effects for specific covariates were reported, the adjusted treatment effects were used.

Due to varying data collection and reporting methods, this review included both continuous and dichotomous scales. Meta-analyses were conducted using Review Manager (RevMan version 5.3) software [16]. It was anticipated that there would be considerable heterogeneity in the data, measured as I^2^, so a random-effects model was applied.

For continuous scales, treatment effects were estimated using standardised mean differences (SMDs). This requires the extraction of mean scores, standard deviations, and sample sizes for each arm. When standard deviations required for the analyses were not available, they were calculated from confidence intervals (CIs), as suggested in the Cochrane handbook [16]. For dichotomous data, treatment effects were estimated using odds ratios by extracting the number of events and sample sizes. All analyses were conducted as planned.

The newly included studies were added to the 9 previous studies in each analysis. Analyses were run for end of treatment and follow-up when available. End of treatment was defined as data collected within 3 months or less from the end of treatment; follow-up was defined as more than 3 months after the end of treatment.

### Quality of studies

The risks of bias were assessed using the Cochrane guidance [16]. Each study was classified for each of the categories into either low risk, high risk or unclear risk, with justifications. This quality assessment was completed by 2 authors independently (AH and AW), and disagreements were resolved by reference to the data in question. We related the risk of bias categories to the interpretation of effect sizes for the outcomes of studies.

## Results

**Fig 1:**
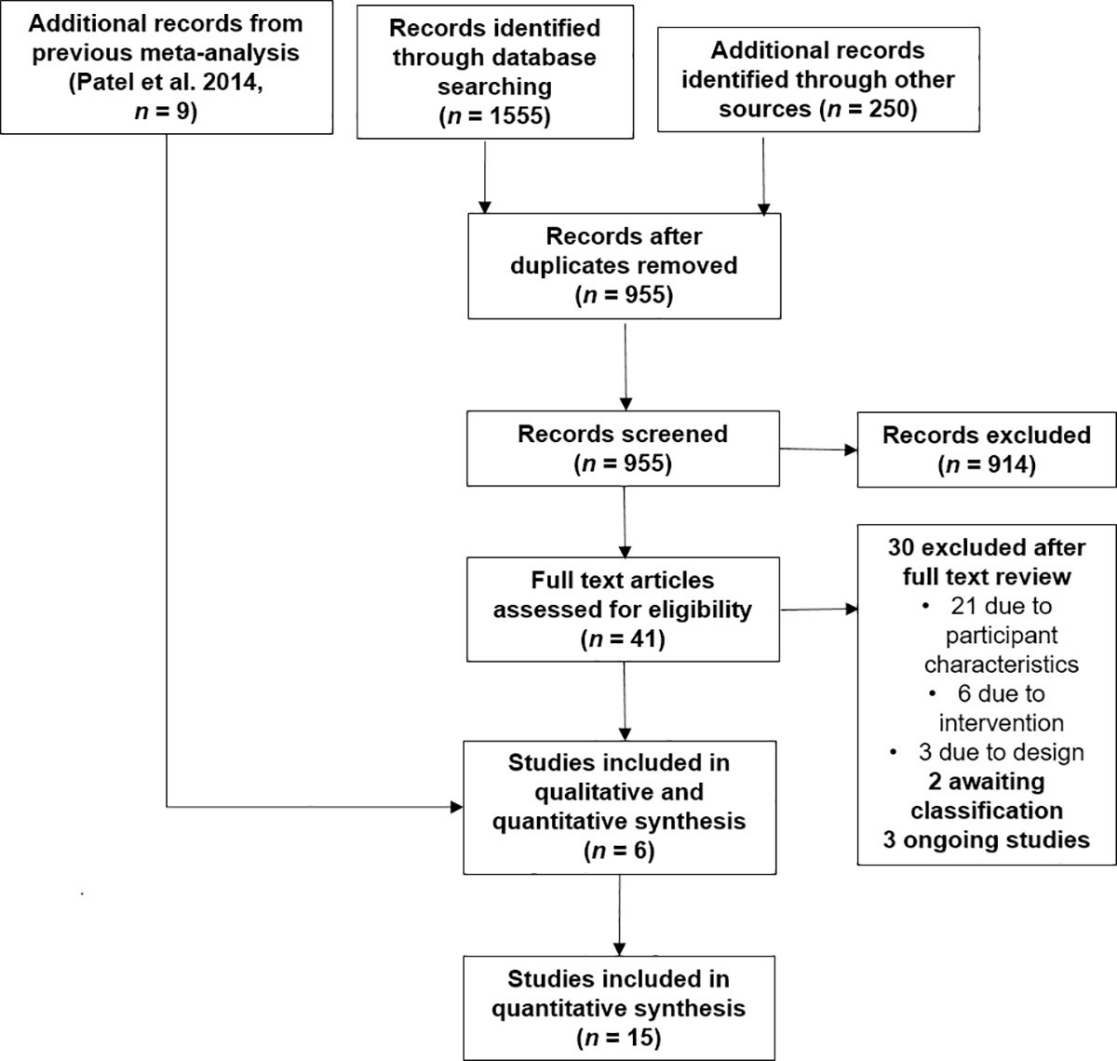
Study flow diagram.

From an initial screen of 1,805 abstracts and titles, 6 RCTs since 2014 met our inclusion criteria [17–22] and were combined with the 9 RCTs identified in the previous meta-analysis (Fig 1) [23–31]. The characteristics of the 15 included studies are summarised in S1 Table. All eligible studies were of psychological interventions. Trials included 1,373 participants at the end of treatment (mean per study = 92) of the 1,585 that started treatment; a mean study completion rate of 86.6% with a range from 50% to 100%. Studies included 589 females and 784 males. Seven trials were conducted in Europe, 5 in Asia, and 3 in Africa. The most commonly used intervention was narrative exposure therapy (4 studies) or testimony therapy (3 studies), both of which draw on creating a testimony of traumatic events. Of the 6 new studies, all provided analysable data after calculating the standard deviation from CIs or standard errors. When neither CIs nor mean scores were available [14,21], the author was contacted, and the mean scores and standard deviations were obtained.

### Quality of studies

**Fig 2:**
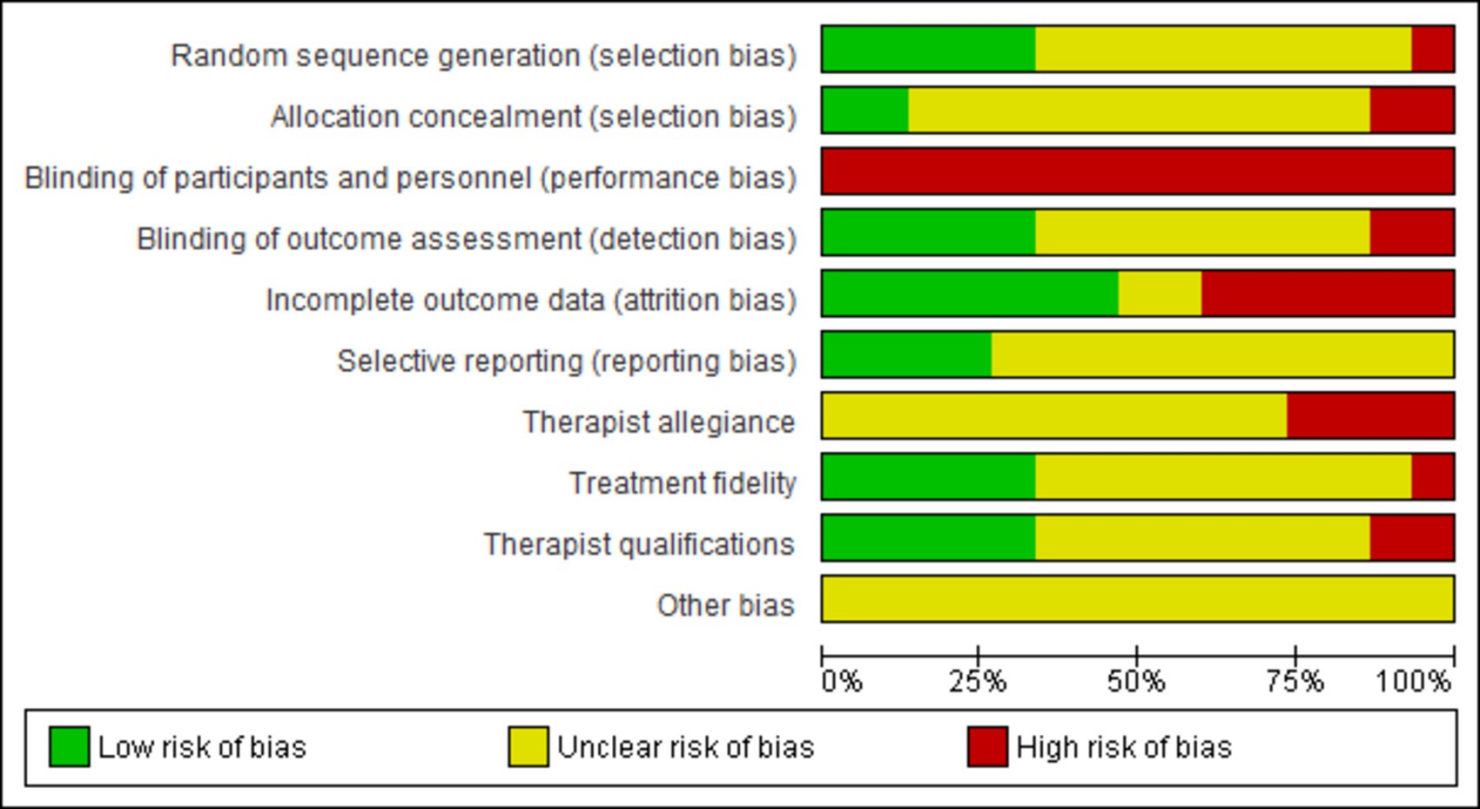
Risk of bias.

According to Cochrane risk of bias assessment [16], one study had a high risk of bias in random sequence generation, 2 had a high risk of bias in allocation concealment, all 15 had a high risk of performance bias (inevitable in psychological treatment trials), 2 had a high risk of detection bias, 6 had a high risk of attrition bias, and no studies had a high risk of reporting bias. Therapist allegiance, treatment fidelity, therapist qualifications, and other biases were also included. Four studies had a high risk of bias due to therapist allegiance, 2 had a high risk of bias due to therapist fidelity, and 2 had a high risk of bias due to therapist qualifications (Fig 2). Other biases included varying content and length of treatment as judged by therapist according to need, as well as the absence of protocol for adaptation and translation of measures. A full breakdown of the risk of bias in each study is available in S1 Table.

### PTSD symptoms

**Fig 3:**
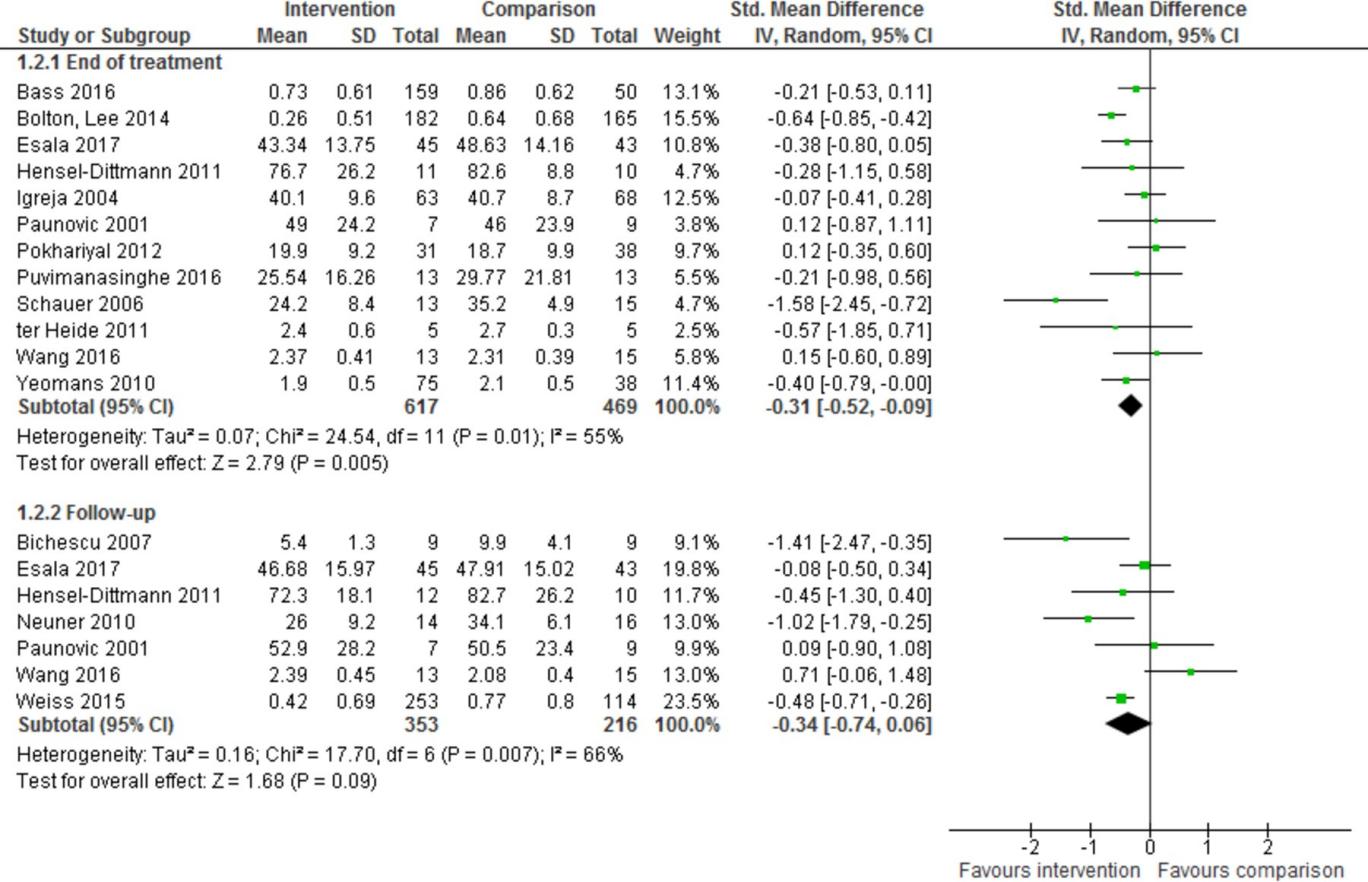
Effect of intervention on PTSD symptoms at end of treatment and follow-up. PTSD, post-traumatic stress disorder.

Twelve trials, with a total of 1,086 participants, reported data for PTSD symptoms no more than 3 months after the end of treatment [17–21,24–31], using several scales but all based on a similar formulation of PTSD. They were analysed for the effect of psychological intervention on PTSD at end of treatment using SMDs (Fig 3). There was a small to moderate reduction in PTSD symptomatology at the end of treatment (SMD −0.31, 95% CI −0.52 to −0.09, *z* = 2.79, *p* = 0.005). Between-study heterogeneity, I^2^, was 55% (95% CI 0.38–0.68), indicating substantial heterogeneity [16]. The confidence in these results is limited overall, as unblinding of assessors may have contributed to detection bias in all but one study [30].

Seven trials, with 569 participants, reported data for PTSD symptoms more than 3 months after the end of treatment [19,21–24,26,27]. All used the Harvard Trauma Questionnaire (HTQ) to measure symptoms with the exception of Esala and Taing [19], who used the PTSD Checklist for the Diagnostic and Statistical Manual of Mental Disorders, Fifth Edition (DSM-5). They were analysed for the effect of psychological intervention on PTSD at follow-up using SMDs (Fig 3).

There was no difference between the intervention group and the control group (SMD −0.34, 95% CI −0.74 to 0.06, *z* = 1.68, *p* = 0.09) in PTSD symptoms at follow-up. Given the large CI, the precision of estimate was low, and all but one study [22] appeared to be underpowered. Heterogeneity was substantial at 66% (95% CI 0.49–0.77).

### PTSD caseness

**Fig 4:**
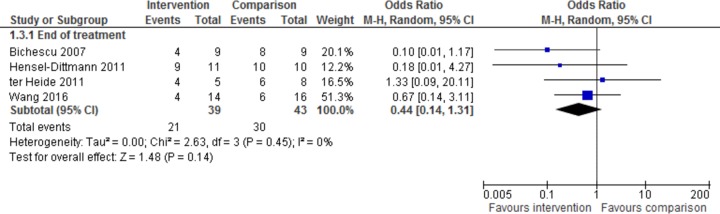
Effect of intervention on PTSD caseness at end of treatment. M-H, Mantel-Haenszel; PTSD, post-traumatic stress disorder.

Four trials with 82 total participants, classifying participants using caseness as meeting criteria for PTSD no more than 3 months after the end of intervention [21,23,24,30], were analysed for the effect of psychological intervention on PTSD caseness at end of treatment (Fig 4). There was no overall benefit, with an odds ratio of 0.44 (95% CI 0.14–1.31, *z* = 1.48, *p* = 0.14). A heterogeneity of I^2^ = 0% was noted for this comparison (95% CI 0–0.61), and a number of sources of bias in methodology were observed.

Only one trial compared PTSD caseness in intervention and control groups, at 6-month follow-up for 28 participants [21]. Caseness was significantly higher at 6-month follow-up in the intervention group compared with the control group, with an odds ratio of 7.58 (95% CI 1.2–48, *z* = 2.15, *p* = 0.03).

### Psychological distress

**Fig 5:**
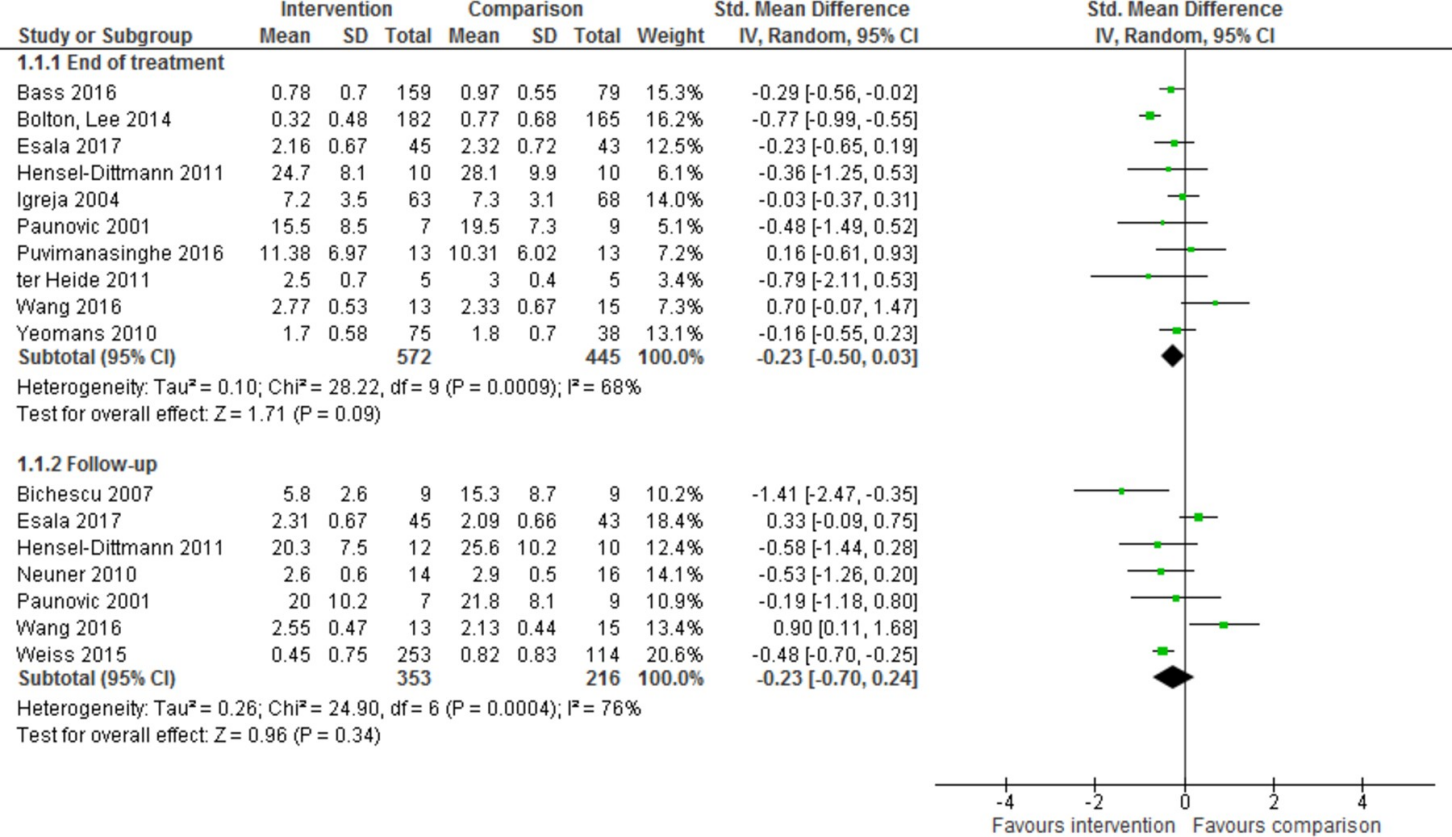
Effect of intervention on psychological distress at end of treatment and follow-up.

Ten trials reported data for psychological distress, measured as depression, no more than 3 months after the end of treatment, with 988 participants [17–22,24,25,27,30]. They were analysed for the effect of psychological intervention on psychological distress at the end of treatment (Fig 5). There was no benefit of treatment over control (SMD −0.23, 95% CI −0.50 to 0.03, *z* = 1.71, *p* = 0.09) with a substantial heterogeneity of I^2^ = 68% (95% CI 0.56–0.77).

Seven trials reported data for psychological distress, measured as depression using the Hopkins Symptom Checklist-25 (HSCL-25), more than 3 months after the end of treatment, with a total of 569 participants [19,21–24,26,27]. They were analysed for the effect of psychological intervention on psychological distress at follow-up using SMDs (Fig 5). There was no benefit of treatment over control for psychological distress at follow-up (SMD −0.23, 95% CI −0.70 to 0.24, *z* = 0.96, *p* = 0.34), and heterogeneity was considerable (I^2^ = 76%, 95% CI 0.65–0.80).

### Functioning

**Fig 6:**
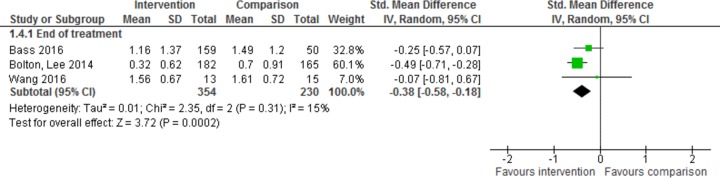
Effect of intervention on functioning at end of treatment.

Three trials reported data for functioning at the end of treatment, for 584 participants [17,18,21], and were analysed for the effect of psychological intervention on functioning at the end of treatment (Fig 6). There was a moderate benefit of intervention over control for functioning (SMD −0.38, 95% CI −0.58 to −0.18, *z* = 3.72, *p* = 0.0002). A heterogeneity of I^2^ = 15% was observed (95% CI 0–0.73).

Only one study (28 participants) provided analysable data showing effects at 6-month follow-up [21] and no statistically significant benefits for treatment over control (SMD 0.63, 95% CI −0.13 to 1.40, *z* = 1.62, *p* = −0.11).

### Quality of life

Two trials [20,30], with 36 participants, assessed quality of life after treatment. Their scales were constructed with opposite direction for improvement; the trial by Puvimanasinghe and Price [20] was reversed so that a positive effect size represented improvement. There was no effect of intervention over control on quality of life (SMD 0.38, 95% CI −0.28 to 1.05, z = 1.14, *p* = 0.26) with a low precision of estimate. No study assessed quality of life at follow-up.

### Adverse events and dropout

Two studies reported on adverse effects of treatment. Weiss and colleagues [22] reported that one participant attempted suicide after the first therapy session. The authors related this to the participant being related to the therapist and the therapist failing to notify the supervisor due to stigma concerns in the family. Another participant was hospitalised with severe depression and received therapy in the hospital but did not return to the study, and one participant died of a heart attack with no apparent relationship to participation in the study. In Wang and colleagues’ [21] study, the intervention group increased in PTSD caseness over follow-up, a statistically significant finding, but the authors were not able to explain this result.

All but 2 trials [23,29] reported dropout during treatment. Of these, 4 reported greater than 20% dropout in the intervention arm [19,24,27,30], and one trial reported a 28% exclusion of participants overall, with no further detail given [28]. Four studies provided detailed reasons for dropout [18,24,27,30].

### Clinical meaning of changes

Calculation of the SMD assumes that differences in standard deviations among studies reflect differences in assessment scales and not real differences in variability among study populations [16]. We chose Wang and colleagues’ study [21] to calculate differences in PTSD symptoms using the HTQ, and in psychological distress (depression) using the HSCL-25. The HTQ uses a 4-point severity response scale. Respondents endorse how much each symptom has bothered them in the past week, as follows: not at all (1), a little bit (2), quite a bit (3), or extremely (4). The total score is the mean of item scores, with 2.5 suggested as the clinical cut-off score, above which a respondent has a high likelihood of PTSD [32]. The small to moderate effect size in reduction of PTSD symptoms for intervention over control represented reduction of the mean pretreatment HTQ score of 2.49 to 2.37 post treatment. That is, participants fell slightly below clinical cut-off both before and after treatment, so the clinical significance of this change is negligible. The HSCL-25 assessed depression, with 1.75 suggested as the clinical cut-off score, with high scores indicating depression. Again relating these scores to the study by Wang and colleagues [21], mean scores at pretreatment assessment (3.02), post-treatment assessment (2.77), and follow-up (2.55) all fell within the clinical range for depression.

## Discussion

This systematic review and meta-analysis of 15 studies of interventions for torture survivors included 1,373 participants from 10 countries. Six of the 15 studies were published since the previous review, but the sample size increased 3-fold. The range of treatments was somewhat wider, but treatments were still most often compared with inactive controls rather than with other treatment. The problems of torture survivors were largely conceptualised in terms of PTSD symptoms that constituted the focus of treatment and, often, the primary outcome. Meta-analysis demonstrated few benefits of treatment: a statistically significant but clinically small decrease in PTSD symptoms at the end of treatment—from varied psychological interventions compared to mostly inactive controls—not found at follow-up. Other outcomes—PTSD caseness, psychological distress, usually depression and often of clinical severity—were not significantly different either at the end of treatment or at follow-up, with the exception of a worsening of PTSD caseness at follow-up, a poorer outcome than in the previous review [12] and clinically very disappointing. Few studies assessed functioning or quality of life, so results must be interpreted with caution, but they showed no improvement in quality of life and only in functioning, at the end of treatment but not at follow-up.

Outcomes representing broader health and participation in society were neglected, as was the context of social, economic, and political uncertainties survivors face; threats to civil and legal status, accommodation, safety, connections with family and friends, and other assaults on well-being [8,33,34]. Because refugees have a high rate of life events that can facilitate or undermine treatment gains, it would be helpful for studies to monitor these changes across the timescale of treatment and follow-up [35]. It was disappointing to find these shortcomings persisting despite comment in our previous review [12] and in others [36,37].

Although it should be interpreted with caution, the finding of worsening at follow-up in the study by Wang and colleagues [21], using CBT with prolonged exposure, should alert researchers to the importance of studying long-term outcomes and the potentially harmful effects of psychological interventions and other contextual factors post treatment. Furthermore, 4 out of the 15 trials reported over 20% dropout in the intervention arm. It is possible that this is a function of greater social instability of the participant population and understandable preoccupation with meeting basic human needs and rights. However, more investigation of treatment expectations and acceptability is required. Conducting and analysing follow-up interviews, using nonaligned and nonbiased interviewers, would lead towards better understanding of what may work and for whom.

Other reviews of psychological treatments for torture survivors [36,38] or for traumatised refugees [39] have produced more optimistic accounts of benefits of therapy, although they raise similar concerns regarding methodology and cultural appropriateness of interventions. By contrast, Salo and Bray [37] reviewed interventions in relation to what they described (drawing on Bronfenbrenner [40]) as the ‘ecological’ needs of torture survivors: microsystem life domain, such as family, social, legal, and occupational domains; macrosystem domain, mainly consisting of cultural and language features of the trials; and the chronosystem domain, represented in time of follow-up assessments. They found relatively scant recognition of needs in any of these areas, either in assessment or intervention. This appears to be a very promising framework for reconsidering therapeutic interventions in the field.

Methodological quality of the included studies was largely similar to that in our previous review. Apart from the absence of blinding of therapists or patients to treatment allocation, rarely possible in trials of psychological treatment, bias arose mainly from incomplete reporting of outcomes, dropping noncompleters from outcome analysis, and uncertainty about whether the intended treatment had been delivered as designed, mainly because of lack of therapist qualifications to deliver it. Whether training volunteer therapists, with no existing clinical competences, in the specific therapeutic techniques for the trial is adequate to produce treatment fidelity is an open question and should be addressed within trials. The same comment applies to cultural adaptation of treatment that originated in Western healthcare. Studies gave little detail of what it was they meant by ‘cultural adaptation’, beyond translation of outcome scales and treatment materials, but effective cultural adaptation involves extensive work between people from all the main cultures represented in a study, who understand the context and content of treatment. Similar methods are required for true validation of translated scales in the languages of the cultures in which they will be used [41]. Even when these procedures are followed, it is by no means clear how a treatment is established as culturally adapted beyond the claims of its authors.

The review has some limitations that potentially affect conclusions. Our search could have been widened by including the grey literature, but a zero yield from around 1,500 chapters, reports, and other articles accessed for the previous review decided us against it. It is possible that in the grey literature, or even in the peer-reviewed literature, our relatively broad search nevertheless missed a trial labelled in a way we did not anticipate, since the nomenclature is not well standardised. While we did not exclude studies in other languages, the majority of the databases searched have shown varied and incomplete coverage of non-English material [42] particularly from low- and middle-income countries [43], indicating a potential database coverage bias. A possible further analysis would have been to fit a model to all effect sizes of each outcome, including time (end of treatment versus follow-up) as a moderator; because we did not, we cannot draw conclusions about maintenance of treatment gains at follow-up. We interpreted our findings according to dichotomous notions of statistical significance and recognise that some overall effect sizes could change (for better or worse) with the addition of one or more studies.

Heterogeneity among studies was substantial and arose from multiple sources: participants, therapists, therapeutic methods, outcomes, delivery, and setting. This produced generally high levels of between-study heterogeneity (I^2^) that made estimates of effect sensitive to inclusion or exclusion of single studies. Given the weakness and lack of precision of the I^2^ statistical test [44], we also calculated the CIs as suggested by Higgins and Thompson [45]. While CIs were generally narrow in cases of high heterogeneity, where low heterogeneity was indicated, I^2^ = 0% for PTSD caseness at end of treatment and I^2^ = 15% for functioning at end of treatment, wide CIs were produced ranging from 0% to 61% and 73%, respectively, indicating caution in inferring heterogeneity in these cases. We did not anticipate having the power available for subanalyses, but these could be planned in a further update, to investigate each source of heterogeneity. Although widening our scope to refugee studies would have included some family and community interventions, heterogeneity would likely have been even greater, exacerbating problems of interpretation.

Given the complexity of torture survivors’ needs and the obstacles they face in reconstructing a meaningful life, the emphasis of interventions on symptoms of PTSD is strikingly narrow, unless reducing or resolving these symptoms is seen as a priority or as the key to other improvements; none of the studies asserted this. It is not even clear that basic security and financial needs are addressed before offering specialised psychological interventions [46]. Thus, integration of interventions addressing the needs and priorities of torture survivors (not assessed or stated) seemed largely lacking. Recruitment into the trial was assumed to mean that survivors’ PTSD symptoms were their priority as the target of intervention, though the fact that 4 out of 15 included studies reported greater than 20% dropout in the intervention arm raises questions about the relevance and appropriateness of the interventions for survivors. Furthermore, the development of interventions in terms of cultural and language appropriateness may require more fundamental exploration and questioning of Western models of psychological problems and treatment than was evident in these trials. Recent models of collaborative care [47] go some way towards this but still fall short of the ecological scope described by Salo and Bray [37]. Last, where resources are scarce and far outstripped by needs, as in many low- and middle-income countries, the model of training local volunteers or healthcare staff in Western methods of intervention delivered mostly as individual therapy may mean that interventions are more culturally embedded (depending on what the trainers or study researchers allow by way of adaptation). This, however, needs empirical support, as well as an assessment of the potential harmful impacts on the volunteer therapists and on (other) survivors they work with.

Perhaps it is the limitation of this review to RCTs that means that the newer trials largely resembled the older ones, except in combining a wider range of interventions; there was little evidence of more collaborative and integrated interventions such as those developing for refugee populations [48] or envisaged in a social-ecological framework [37]. It might be that single case methods [49] are more applicable to assessing psychological, social, welfare, and other interventions for the complex and diverse needs of many torture survivors, for whom distress stems not only from the violent and traumatic experiences endured but also from current social, material, and legal conditions [34].

Evaluation of interventions needs to match this breadth of difficulties and at minimum interventions require addressing quality of life and follow-up over realistic time frames. Qualitative studies could helpfully inform more participant-focused assessment of treatment outcome with the addition of observed events such as improved overall health; enrolment in further education, training, or work; and participation in community or society.

In conclusion, all RCTs we found in this systematic review and meta-analysis were of psychological interventions. Small improvements for intervention over control were found for PTSD symptoms and functioning after treatment but not at follow-up, nor was any improvement evident for psychological distress at either time point or for quality of life at the end of treatment. The overall confidence in these results and precision of estimate is still less than satisfactory, and further studies are likely to change the estimates of effect, but the differences between our findings and the impression of treatment effectiveness from narrative reviews are substantial and suggest that more survivor-focused conceptualisation of problems and improved methodology are needed.

## Supporting information

S1 PRISMA Checklist PRISMA, Preferred Reporting Items for Systematic Reviews and Meta-Analyses. (DOC)

S1 Text Systematic review protocol. (DOCX)

S1 Table Characteristics of included studies. CMHW, community mental health worker; NET, narrative exposure therapy.
(DOCX)

## Abbreviations

CBT: cognitive behavioural therapy
CI: confidence interval
DSM-5: Diagnostic and Statistical Manual of Mental Disorders, Fifth Edition
HSCL-25: Hopkins Symptom Checklist-25
HTQ: Harvard Trauma Questionnaire
PRISMA: Preferred Reporting Items for Systematic Reviews and Meta-Analyses
PTSD: post-traumatic stress disorder
RCT: randomised controlled trial
SMD: standardised mean difference
