# Supplementary material for: Psychological, social, and welfare interventions for torture survivors: A systematic review and meta-analysis of randomised controlled trials
Source: PLoS Med. 2019 Sep 24;16(9):e1002919. doi: 10.1371/journal.pmed.1002919 (PMC6759153; doi:10.1371/journal.pmed.1002919)
Supplement: S1 Text — (DOCX) [file pmed.1002919.s002.docx]

**S1 Text. Systematic Review Protocol**

**Psychological, social, and welfare interventions for torture survivors: a systematic review and meta-analysis of randomised controlled trials**

Aseel Hamid*, Nimisha Patel & Amanda C. de C. Williams

[*aseel.hamid@ucl.ac.uk](mailto:*aseel.hamid@ucl.ac.uk)

**Introduction**

In 2014, torture and ill treatment has been reported in at least 141 countries [1]. Long-standing and ongoing armed conflict has likely led to the increased use of torture since then. In 2014, a Cochrane systematic review [2] aimed to summarise psychological, social, and welfare interventions and found only eligible randomised controlled trials (RCTs) of psychological treatment. These studies showed no immediate benefits for psychological therapy for psychological distress (as measured by depression symptoms), post-traumatic stress disorder (PTSD) symptoms, PTSD caseness or quality of life. At follow-up, four studies showed moderate effect sizes in reducing psychological distress and PTSD symptoms. Conclusions were tentative, given the low quality of evidence, with underpowered studies and outcomes assessed in non-standard ways.

More recently in 2017, a meta-analysis of interventions of survivors of mass violence in low and middle-income countries showed a large improvement in PTSD and depression across treatment [3]. Another review conducted in 2016 concluded that cognitive behavioural therapy (CBT) interventions produced the best treatment outcome for PTSD and/or depression. However, both reviews recruited more widely than torture survivors and no recent systematic reviews or meta-analyses have focussed on interventions for torture survivors.

This systematic review aims to update the previous review conducted in 2014 to assess the reported benefits or adverse outcomes in the domains of PTSD symptoms, PTSD caseness, psychological distress, functioning and quality of life for psychological, social and welfare interventions for torture survivors.

**Methods**

This review will follow the PRISMA standards for conducting systematic reviews and meta-analyses, following the same protocol as in Patel and colleagues’ review [2].

**Inclusion criteria**

Studies will be included if they:

- employed randomised controlled methodology
- included male and female participants of all ages and ethnicities
- included at least 50% of the sample as survivors of any type of torture, as defined by the study researchers, regardless of diagnosis
- included psychological interventions, social interventions or welfare interventions
- included interventions undertaken with individuals or with families or groups
- had control comparisons, including waiting list, no treatment, (access to) standard care, attention control or alternative treatment that was likely to engender expectations of improvement in those allocated to that condition, such as psycho-education or peer support
- measured psychological distress as a primary outcome
- measured any of the following as a secondary outcome:
  - change in psychological status (usually by way of change in symptoms or diagnostic category) or change in target behaviour (at a group or individual level)
  - change in quality of life or well-being
  - change in participation and functioning by way of engagement in education, training, work or community activity
  - change of quality and/or quantity of family or social relationships
  - other-reported psychological functions (others include clinicians, parents and teachers)
  - use of health care resources
  - ratings of the intervention itself (such as satisfaction with intervention and therapeutic alliance)
- measured outcomes immediately post treatment and/or up to around 3 months after the end of treatment, and if multiple follow-ups are used, the longest up to one-year will be used

Where more than one outcome measure was included pertaining to the same domain of interest, and all described the domain adequately, primary preference will be given to a measure also used in other studies included in the previous review. Secondary preference will be given to any measure that authors stated was tested for suitability in the population included in the respective trial. Where there are poster abstracts, missing data or unpublished studies and contact details available, authors are to be contacted for clarification.

**Search strategy**

Using a highly sensitive search (see search terms below) the following searches will be conducted.

*Electronic searches*

1. OVID PsycINFO
2. OVID Medline
3. OVID Embase
4. Cochrane Central Register of Controlled Trials (CENTRAL)
5. ClinicalTrials.gov
6. WHO International Clinical Trials Registry Platform
7. Cumulative Index to Nursing and Allied Health Literature (CINAHL)
8. Web of Science
9. PTSDpubs (previously known as ProQuest Published International Literature on Traumatic Stress; PILOTS)

*Searching other resources*

1. Danish Institute against Torture (DIGNITY; 1st January 2014 to 22nd June 2019)
2. Reference lists of reviews emerging from the searches
3. Reference lists from the final set of included studies
4. Contact with authors for which a reference was found but was not yet published

**Search terms**

The list below contain search terms for respective databases.

*OVID PsycINFO (1^st^ January 2014 to 22^nd^ June 2019)*

A sensitive search was conducted using terms for population (only) plus an RCT filter:

1. torture/

2. torture$.mp.

3. ‘prisoners of war’/

4. ((trauma* or psychotrauma* or violence) and (asylum$ or refugee$ or hostage$)).mp.

5. ((organi#ed or mass) adj3 violence).mp.

6. exp survivors/ and War/

7. ((surviv$ or victim*) adj7 war).mp.

8. (polit$ adj7 persecut$).mp.

9. exp genocide/

10. genocide.mp.

11. or/1-10

12. clinical trials.sh.

13. mental health program evaluation.sh.

14. treatment effectiveness evaluation.sh.

15. placebo.sh.

16. placebo$.ti,ab.

17. (wait* and list* and (control* or group)).ab.

18. (‘treatment as usual’ or TAU).ab.

19. randomly.ab.

20. randomi#ed.ti,ab.

21. trial.ti,ab.

22. ((singl$ or doubl$ or trebl$ or tripl$) adj3 (blind$ or mask$ or dummy)).mp.

23. (control$ adj3 (trial$ or study or studies or group$)).ti,ab.

24. factorial$.ti,ab.

25. allocat$.ti,ab.

26. assign$.ti,ab.

27. (crossover$ or cross over$).ti,ab.

28. (quasi adj (experimental or random$)).mp.

29. ‘2000’.md.

30. or/12-29

31. 11 and 30

*OVID MEDLINE (1^st^ January 2014 to 22^nd^ June 2019)*

1. torture/

2. torture$.tw.

3. ((trauma* or psychotrauma* or violence) and (asylum$ or refugee$ or hostage$)).mp.

4. ((organi#ed or mass) adj3 violence).tw.

5. exp survivors/ and war/

6. ((surviv$ or victim*) adj7 war).tw.

7. (polit$ adj7 persecut$).tw.

8. genocide.tw.

9. or/1-8

10. randomized controlled trial.pt.

11. controlled clinical trial.pt.

12. randomi#ed.ti,ab.

13. randomly.ab.

14. placebo.ab.

15. (wait* and list* and (control* or group)).ab.

16. (treatment as usual or TAU).ab.

17. trial.ab.

18. groups.ab.

19. (control$ adj3 (trial or study)).ab,ti.

20. ((singl$ or doubl$ or tripl$ or trebl$) adj3 (blind$ or mask$ or dummy)).mp.

21. or/10-20

22. 9 and 21

*OVID EMBASE (1^st^ January 2014 to 22^nd^ June 2019)*

1. torture/

2. torture$.tw.

3. torture survivor/

4. ((trauma* or psychotrauma* or violence) and (asylum$ or refugee$ or hostage$)).mp.

5. ((organi#ed or mass) adj3 violence).tw.

6. survivor/ and war/

7. ((surviv$ or victim*) adj7 war).tw.

8. (polit$ adj7 persecut$).tw.

9. genocide.mp.

10. or/1-9

11. randomized controlled trial.de.

12. randomization.de.

13. randomi#ed.ti,ab.

14. randomly.ab.

15. placebo.de.

16. placebo$.ti,ab.

17. (wait* and list* and (control* or group)).ab.

18. (treatment as usual or TAU).ab.

19. ((singl$ or doubl$ or trebl$ or tripl$) adj3 (blind$ or mask$ or dummy)).mp.

20. factorial$.ti,ab.

21. allocat$.ti,ab.

22. assign$.ti,ab.

23. volunteer$.ti,ab.

24. crossover procedure.de.

25. (crossover$ or cross over$).ti,ab.

26. (quasi adj (experimental or random$)).mp.

27. (control$ adj3 (trial$ or study or studies or group$)).ti,ab.

28. or/11-27

29. 10 and 28

*CENTRAL (1^st^ January 2014 to 22^nd^ June 2019)*

#1 MeSH descriptor Torture explode all trees

#2 torture*

#3 (trauma* or psychotrauma* or violence) and (asylum* or refugee* or hostage*)

#4 (organized or organised or mass) and violence

#5 MeSH descriptor War this term only

#6 MeSH descriptor Survivors explode all trees

#7 (#5 AND #6)

#8 (surviv* or victim*) and war

#9 (polit* and persecut*)

#10 genocide

#11 (#1 OR #2 OR #3 OR #4 OR #7 OR #8 OR #9 OR #10)

*ClinicalTrials.gov and WHO ICTRP (1^st^ January 2014 until June 2019)*

Tortur*

*CINAHL-EBSCO Host (1^st^ January 2013 until 22^nd^ June 2019)*

S1 (MH “Torture”)

S2 (MH “Torture Survivors”)

S3 (torture*)

S4 ((trauma* or psychotrauma* or violence) and (asylum* or refugee* or hostage*))

S5 ((organi#ed or mass) N3 violence)

S6 (MH “Survivors”) and (MH “War+”)

S7 ((surviv* or victim*) N7 war)

S8 (polit* N7 persecut*)

S9 (genocide)

S10 (S1 or S2 or S3 or S4 or S5 or S6 or S7 or S8 or S9)

S11 (MH “Clinical Trials+”)

S12 (PT Clinical trial)

S13 (TX clini* N1 trial*)

S14 (TX ((singl* N1 blind*) or (singl* N1 mask*)) or TX ((doubl* N1 blind*) or (doubl* N1 mask*))

or TX ((tripl* N1 blind*) or (tripl* N1 mask*)) or TX ((trebl* N1 blind*) or (trebl* N1 mask*)))

S15 (TX randomi* control* trial*)

S16 (MH “Random Assignment”)

S17 ((TX random* allocat*) or (TX allocat* random*))

S18 (TX placebo*)

S19 (TX (wait* and list* and (control* or group)))

S20 ((TX “treatment as usual”) or (TX TAU))

S21 (TX (control* N3 (trial* or study or studies or group*)))

S22 (MH “Quantitative Studies”)

S23 (S11 or S21 or S13 or S14 or S15 or S16 or S17 or S18 or S19 or S20 or S21 or S22)

S24 (S10 and S23)

*Web of Science (1^st^ January 2013 to 22^nd^ June 2019)*

1. torture*

2. ((trauma* or psychotrauma* or violence) and (asylum* or refugee* or hostage*))

3. ((organized or organised or mass) NEAR/3 violence)

4. ((surviv* or victim*) NEAR/7 war)

5. (polit* NEAR/7 persecut*)

6. genocide

7. (#6 OR #5 OR #4 OR #3 OR #2 OR #1)

8. (randomized or randomised)

9. (random* NEAR/3 (allocat* or assign*))

10. placebo*

11. ((wait* and list*) SAME (control* or group))

12. (“treatment as usual” or TAU)

13. (control* NEAR/3 (trial or study))

14 ((singl* or doubl* or tripl* or trebl*) NEAR/3 (blind* OR mask* OR dummy))

15. (quasi NEAR/3 (experimental or random*))

16. (#15 OR #14 OR #13 OR #12 OR #11 OR #10 OR #9 OR #8)

17. (#7 and #16)

*PTSDpubs (previously known as PILOTS-ProQuest Host; 1^st^ January 2013 to June 2019)*

Randomised AND tortur*

randomized AND tortur*

randomly allocated AND tortur*

randomly assigned AND tortur*

quasi-random AND tortur*

quasi-randomized AND tortur*

quasi-randomised AND tortur*

placebo AND tortur*

controlled trial AND tortur*

controlled study AND tortur*

*Danish Institute against Torture (DIGNITY; 1^st^ January 2014 to 22^nd^ June 2019)*

Randomised AND tortur*

randomized AND tortur*

randomly allocated AND tortur*

randomly assigned AND tortur*

quasi-random AND tortur*

quasi-randomized AND tortur*

quasi-randomised AND tortur*

placebo AND tortur*

controlled trial AND tortur*

controlled study AND tortur*

**Data extraction**

A data extraction table was designed on the basis of the previous review to document the following study details:

- Study design
- Setting of intervention
- Types of interventions
- Intervention protocol
- Sample size at baseline and outcome assessments
- Baseline characteristics of the sample (age, gender, nationality, ethnicity, type of torture experienced, legal status if refugees and asylum seekers, living situation, separation from
- close family members)
- Baseline measures
- Types of practitioners/therapists
- Language/s of assessment; translation, interpretation
- Properties of baseline measures (language, translation, validity)
- Outcome measures at end of intervention(s) and at follow up assessment.
- Completion rates
- Adherence to, participation in treatment

The following criteria, based on Cochrane guidance [5] will be used as indicative of risk of bias:

- Random sequence generation (selection bias)
- Allocation concealment (selection bias)
- Blinding of participants and personnel (performance bias)
- Blinding of outcome assessment (detection bias)
- Incomplete outcome data (attrition bias)
- Selective reporting (reporting bias)
- Therapist allegiance
- Treatment fidelity
- Therapist qualifications
- Other bias not included in above categories

**Data analysis**

Where available, data will be extracted from included studies for the following outcomes: PTSD symptoms, PTSD caseness, psychological distress (measured as depressive symptoms), functioning and quality of life. These outcomes will be extracted for the end of treatment (defined as collected within three months or less of the end of intervention) and at follow-up (more than three months after the end of treatment) where available. RevMan 5 software will be used to conduct a meta-analysis, applying a random-effects model given the various sources of diversity likely to occur. Data will be displayed graphically and hierarchically in forest plots.

**References**

[1] Amnesty International. Torture in 2014: 30 years of broken promises. Amnesty International; 2014 (http://www.amnesty.org/en/library/info/ACT40/004/2014/en; accessed 21 June 2019).

[2] Patel N, Kellezi B, de C Williams AC. Psychological, social and welfare interventions for psychological health and well‐being of torture survivors. Cochrane Database Syst Rev. 2014;(11): Art. No.: CD009317.

[3] Morina N, Malek M, Nickerson A, Bryant RA. Meta‐analysis of interventions for posttraumatic stress disorder and depression in adult survivors of mass violence in low‐and middle‐income countries. Depress Anxiety. 2017 Aug;34(8):679-91.

[4] Weiss WM, Ugueto AM, Mahmooth Z, Murray LK, Hall BJ, Nadison M, et al. Mental health interventions and priorities for research for adult survivors of torture and systematic violence: a review of the literature. Torture. 2016;26(1):17-44.

[5] Higgins JP. Cochrane handbook for systematic reviews of interventions. Version 5.1. 0 [updated March 2011]. The Cochrane Collaboration. www.cochrane-handbook.org 2011.
