# Supplementary material for: Psychological, social, and welfare interventions for torture survivors: A systematic review and meta-analysis of randomised controlled trials
Source: PLoS Med. 2019 Sep 24;16(9):e1002919. doi: 10.1371/journal.pmed.1002919 (PMC6759153; doi:10.1371/journal.pmed.1002919)
Supplement: S1 Table — CMHW, community mental health worker; NET, narrative exposure therapy. (DOCX) [file pmed.1002919.s003.docx]

**S1 Table. Characteristics of included studies (in alphabetical order)**

**Bass et al. 2016 [1]**

| Methods | **Study design**: randomised controlled trial |
| --- | --- |
| Participants | 209 adults who have experienced or witnessed physical torture  **Diagnosis:** Depression  **Method of diagnosis:** reporting at least 20 on Hopkins Symptom Checklist (HSCL-25) depression scale and meeting criteria in the Diagnostic and Statistical Manual of Mental Disorders (DSM-IV) for major depression.  **Age:** mean age 40 years (range 18 to 82)  **Gender**: 66% male, 34% female  **Location**: Dohuk, Kurdistan, Iraq |
| Interventions | Participants were randomly assigned to:   1. Experimental arm (*n* = 159)   **Duration**: 6-12 sessions depending on client need  **Treatment protocol**: supportive counselling  **Practitioners**: trained community mental health workers (CMHWs) who were permanent employees of the Ministry of Health   1. Comparator arm (*n* = 50)   **Duration**: 3-5 months  **Treatment protocol**: waitlist control: monthly telephone calls to participants for brief check-in  **Practitioners**: same as above |
| Outcomes | **Time points for assessment**: baseline and 1 month post-completion for intervention participants or 3-5 months after baseline for waitlist participants  **Assessment language**: Kurdish  **Primary outcome:** PTSD using the Harvard Trauma Questionnaire (HTQ)  **Secondary outcome**: depressive symptoms and anxiety symptoms (HSCL-25), functional impairment (locally developed scales described elsewhere) and traumatic grief (Inventory of Traumatic Grief) |
| Baseline Characteristics | 20% self-reported disability, majority married, approximately 50% unemployed, and more than 40% reported no education. Demographic characteristics of the participants across the two arms were comparable, with no statistically significant differences. |
| Adherence and Completion | Of the 159 allocated to treatment, 5 never initiated counselling. Of the 154 who initiated counselling, 147 (95.5%) completed treatment. Mean number of sessions attended was 11.29 (range 6-12). 10 individuals in intervention arm were lost to follow up and 7 in comparator arm were lost to follow-up. In total, 188 individuals (90% completed follow up) |
| Notes | The HSCL-25, HTQ and Inventory for Traumatic Grief were adapted and validated for the local context, detailed methods were described elsewhere |
| ***Risk of bias*** | |
| **Bias** | **Author’s judgement and support for judgement** |
| Random sequence generation (selection bias) | **Low risk**. Used random number generation using Stata |
| Allocation concealment (selection bias) | **Unclear risk.** Designation of intervention or waitlist control status was stapled to the back of consent forms |
| Blinding of participants and personnel (performance bias) | **High risk.** Not possible to render participants nor practitioners blind to allocation |
| Blinding of outcome assessment (detection bias) | **High risk.** 82% of the follow-up interviews were blinded |
| Incomplete outcome data (attrition bias) | **Low risk**. Over 90% completed treatment and follow-up, multiple imputation methods were used for incomplete data |
| Selective reporting (reporting bias) | **Low risk.** All scales were reported |
| Therapist allegiance | **Low risk**. All CMHWs from varying health backgrounds and received training in supportive counselling at same time |
| Treatment fidelity | **High risk.** Counselling was given according to need with no specific protocol followed |
| Therapist qualifications | **High risk.** Community based providers were trained for the study but with no previous formal mental health training |
| Other bias | **Unclear risk.** The adaptation of scales and translation was not described |

**Bichescu et al 2007 [2]**

| Methods | **Study design**: randomised controlled trial |
| --- | --- |
| Participants | 18 former political detainees under communist Romania, living at home  **Diagnosis**: PTSD on 2 occasions 1 year apart; no signs of disability on Mini Mental State Inventory  **Method of diagnosis**: Composite International Diagnostic Interview  **Age**: mean 69 years  **Gender**: 94% men, 6% women  **Location**: Romania |
| Interventions | Participants were randomly assigned to:   1. Experimental arm (*n* = 9)   **Duration**: 5 2-hour sessions  **Treatment protocol**: Narrative exposure therapy (NET)  **Practitioners**: Romanian-speaking female PhD psychology student; therapy in own language   1. Comparator arm (*n* = 9)   **Duration**: 1 session  **Treatment protocol**: psychoeducation ; “standardized treatment”  **Practitioners**: Romanian-speaking female PhD psychology student; therapy in own language |
| Outcomes | **Time points for assessment**: pretreatment and at 6-month follow-up  **Assessment language**: Romanian; measures translated as necessary  **Primary outcome:** Symptoms of PTSD (Composite International Diagnostic Interview) for diagnosis and symptom count, no information about validation  **Secondary outcome**: Depression (Beck Depression Inventory) through interview with translation from English |
| Baseline Characteristics | Mean number of mistreatments 13; no detail. Mean of 42 years since release from imprisonment; mean duration of imprisonment 6 years. Education, occupational status and marital status recorded |
| Adherence and Completion | All 18 completed treatment and follow-up |
| Notes | Date of study: 2003. Funding source: Hans-Böckler Foundation and Deutsche Forschungsgemeinschaft. Declarations of interest among primary researchers: no declaration. Assessment by clinical psychology and MA psychology students who were intended to be blind to treatment, which was not entirely successful. |
| ***Risk of bias*** | |
| **Bias** | **Author’s judgement and support for judgement** |
| Random sequence generation (selection bias) | **High risk**. By “random selection procedure of participants’ name-cards”: unclear who performed selection |
| Allocation concealment (selection bias) | **Unclear risk.** No information provided |
| Blinding of participants and personnel (performance bias) | **High risk.** Not possible to render participants nor practitioners blind to allocation. Expectations of benefit not assessed |
| Blinding of outcome assessment (detection bias) | **Unclear risk.** Most blind assessors were arranged, but “it was not possible for us to achieve complete blindness in all cases” as participants revealed details of treatment that identified the condition |
| Incomplete outcome data (attrition bias) | **Low risk**. All participants included: no attrition |
| Selective reporting (reporting bias) | **Unclear risk.** Two measures used and reported: no protocol available |
| Therapist allegiance | **High risk**. Allegiance to NET |
| Treatment fidelity | **Unclear risk.** No information |
| Therapist qualifications | **Unclear risk.** In training |
| Other bias | **Unclear risk.** Real-time translation of assessment measures, so not standardised |

**Bolton et al 2014 [3]**

| Methods | **Study design**: randomised controlled trial |
| --- | --- |
| Participants | 347 displaced Burmese adults who have experienced or witnessed physical torture, imprisonment and “related traumas”  **Diagnosis**: Moderate to severe depression or PTSD  **Method of diagnosis:** reporting at least 20 on the HSCL-25 depression scale while meeting DSM-IV criteria for major depression, and the HTQ for depression and PTSD respectively  **Age:** mean 35.6 years  **Gender**: 63% female  **Location**: Thailand |
| Interventions | Participants were randomly assigned to:   1. Experimental arm (*n* = 182)   **Duration**: 7-13 weekly sessions, average number of sessions: 9.7  **Treatment protocol**: common elements approach, a transdiagnostic treatment developed by the authors  **Practitioners**: trained lay workers, all Burmese refugees who received regular supervision   1. Comparator arm: (*n* = 165)   **Duration**: 3-4 months  **Treatment protocol**: waitlist control: monthly telephone calls  **Practitioners**: project coordinator (no further details given) |
| Outcomes | **Time points for assessment**: baseline and post-completion  **Assessment language**: Burmese  **Primary outcome:** depression and anxiety symptoms (HSCL-25) and PTSD (HTQ)  **Secondary outcome**: aggression behaviour (12 item Aggression Questionnaire), functional impairment (locally developed scales described elsewhere) and alcohol use (Alcohol Use Disorders Identification Test) |
| Baseline Characteristics | “Baseline anxiety was identified as the only measured variable likely to be different between the two groups at baseline and was included in adjusted models” |
| Adherence and Completion | 79% of all participants were followed-up. For the intervention arm, 5 were recruited in error in that they did not meet depression or PTSD criteria, 34 were lost to follow-up in the intervention arm with 18 withdrawing due to lack of time or return to Burma, 1 passed away and 15 could not be located. Of 39 controls lost to follow-up, 8 no longer had time to left the area and 31 could not be located |
| Notes | The mean baseline for all participants for both depression and PTSD was low overall and so researchers are not investigating a highly affected sample. The paper did not include type of traumatic event as a descriptive variable but upon following up with the authors, it was confirmed that 54% of participants reported torture. All outcome measures were adapted to the local context and tested during a prior instrument validation study (described elsewhere) |
| ***Risk of bias*** | |
| **Bias** | **Author’s judgement and support for judgement** |
| Random sequence generation (selection bias) | **Low risk**. Project site director generated random identification numbers using Stata |
| Allocation concealment (selection bias) | **Low risk.** Counsellors opened a pre-sealed envelope (corresponding to the identification number) containing an assignment to treatment or waitlist |
| Blinding of participants and personnel (performance bias) | **High risk**. Not possible to render participants nor practitioners blind to allocation |
| Blinding of outcome assessment (detection bias) | **Low risk**. Baseline and outcome assessors were blind to condition |
| Incomplete outcome data (attrition bias) | **High risk**. More than 20% attrition; those lost to follow-up had higher baseline alcohol use, reported more current problems and were more likely to be non-Burman |
| Selective reporting (reporting bias) | **Unclear risk**. All measures were used and reported, validity and reliability tests reported, the development and adaptation of scales were also reported but no protocol reported and no note on translation |
| Therapist allegiance | **Unclear risk**. All community based providers received training in common elements approach at same time and were of varying backgrounds |
| Treatment fidelity | **Unclear risk**. Authors noted that counsellors implemented common elements approach with fidelity according to supervision reports, but no details of report were given |
| Therapist qualifications | **High risk**. Community based providers, with only 2 of 11 having prior general counselling experience |
| Other bias | **Unclear risk.** Treatment varied across participants according to therapist’s judgement of need |

**Esala and Taing 2017 [4]**

| Methods | Study design: pilot randomised controlled trial |
| --- | --- |
| Participants | 88 Khmer Rouge torture survivors  **Diagnosis**: Moderate to severe depression or PTSD  **Method of diagnosis**: HSCL-25 for depression, Posttraumatic Stress Disorder Checklist for PTSD.  **Age**: mean age 60.4 years  **Gender**: 74% female  **Location**: Cambodia |
| Interventions | Participants were randomly assigned to:   1. Experimental arm (*n* = 45)   **Duration**: 5 days  **Treatment protocol**: testimony therapy plus ceremony  **Practitioners**: counsellors trained in testimony therapy plus ceremony from a German clinician, with biweekly supervision from psychologist in Cambodia.   1. Comparator arm(*n* = 43)   **Duration**: 5 days  **Treatment protocol**: waitlist control: no information available  **Practitioners**: no information available |
| Outcomes | **Time points for assessment**: baseline, 3 months and 6 months  **Assessment language**: Khmer  **Primary outcome**: PTSD (Posttraumatic Stress Disorder Checklist)  **Secondary outcome**: depression and anxiety (HSCL-25). |
| Baseline Characteristics | Over 60% in both groups were married, and the majority (at least 95%) in both groups were Buddhist. 73.3% and 72.1% of the treatment and comparator arm participants respectively are able to read |
| Adherence and Completion | 60 participants were randomised to each group. Of the individuals randomised to the treatment group, 15 chose not to complete baseline or participate in intervention and 9 did not complete at least one of the follow-up assessments. Of the individuals in the comparator arm, 17 participants did not complete the baseline and 3 did not complete at least one of the follow-up assessments |
| Notes | Testimony plus ceremony was culturally adapted for local context |
| ***Risk of bias*** | |
| **Bias** | **Author’s judgement and support for judgement** |
| Random sequence generation (selection bias) | **Unclear risk**. Method of randomisation not described |
| Allocation concealment (selection bias) | **Unclear risk**. Method of allocation not described |
| Blinding of participants and personnel (performance bias) | **High risk.** Not possible to render participants nor practitioners blind to allocation |
| Blinding of outcome assessment (detection bias) | **Low risk.** Measures were administered by a blind assessor at baseline, 3 months and 6 months |
| Incomplete outcome data (attrition bias) | **High risk**. There was moderate to high dropout and no attempt at intention-to-treat analyses |
| Selective reporting (reporting bias) | **Low risk.** All assessment instruments were reported |
| Therapist allegiance | **Unclear risk.** Counsellors prior experience, training and qualifications not reported |
| Treatment fidelity | **Unclear risk**. Counsellors monitored adherence to and deviation from methods |
| Therapist qualifications | **Unclear risk.** Counsellors’ experience and qualifications not reported |
| Other bias | **Unclear risk.** |

**Hensel-Dittman et al 2011 [5]**

| Methods | Study design: randomised controlled trial |
| --- | --- |
| Participants | 28 clinic outpatients, refugees from various countries-most still seeking asylum  **Diagnosis:** PTSD  **Method of diagnosis:** DSM-IV  **Age:** not given, but no differences between groups  **Gender:** not given, but no differences between groups  **Location:** Germany |
| Interventions | Participants were randomly assigned to:   1. Experimental arm (*n* = 15)   **Duration:** 10 individual sessions of mean 90 minutes  **Treatment protocol:** NET (manualised)  **Practitioners:** trained, with interpreter when necessary (17/28)   1. Comparator arm (*n* = 13)   **Duration:** 10 individual sessions of mean 90 minutes  **Treatment protocol:** stress inoculation training, avoiding any element of exposure  **Practitioners:** trained therapists, with interpreter when necessary (17/28), same for both arms |
| Outcomes | **Time points for assessment:** pretreatment and at 6-month and 1-year follow-up  **Assessment language:** measures in German; no information on cross-cultural use  **Primary outcome:** PTSD severity score (clinician-administered scale IV)  **Secondary outcome:** PTSD diagnosis: DSM-IV, Depression: Hamilton Depression Scale |
| Baseline Characteristics | 76% had been tortured; remainder had experienced war. No differences between groups in length of time in Germany, area of origin, education or co-morbid psychiatric disorders, but no baseline data given |
| Adherence and Completion | 5 dropouts NET, 2 dropouts in stress inoculation training (1 stress inoculation training participant deported) |
| Notes | **Date of study:** 2004 to 2007  **Funding source:** European Refugee Fund and Deutsche Forschungsgemeinschaft  **Declarations of interest among primary researchers:** no conflicting interests |
| ***Risk of bias*** | |
| **Bias** | **Author’s judgement and support for judgement** |
| Random sequence generation (selection bias) | **Unclear risk**. Participants matched pairwise by gender, age and region of origin, then allocated by flipping coin |
| Allocation concealment (selection bias) | **Unclear risk**. No information provided |
| Blinding of participants and personnel (performance bias) | **High risk.** Not possible to render participants nor practitioners blind to allocation. Expectations of benefit not accessed |
| Blinding of outcome assessment (detection bias) | **Unclear risk.** Assessors blind (unless accidentally unblinded) to allocation |
| Incomplete outcome data (attrition bias) | **Low risk**. Data provided different N at each time point; analysis by intention-to-treat, so mixed-effect models with neither imputation nor LOCF |
| Selective reporting (reporting bias) | **Unclear risk.** All outcomes reported in trial methods: no protocol available |
| Therapist allegiance | **High risk.** NET: active treatment |
| Treatment fidelity | **Low risk**. Manualised NET |
| Therapist qualifications | **Low risk.** Trained therapists |
| Other bias | **Unclear risk.** Most refugees still had asylum undecided, so may have had an incentive to underreport improvement |

**Igreja et al 2004 [6]**

| Methods | Study design: randomised controlled trial |
| --- | --- |
| Participants | 137 people, post-civil war, mostly rural population seen in their homes  **Diagnosis:** PTSD caseness  **Method of diagnosis:** Self-Inventory for PTSD  **Age:** mean 40 years (SD 14)  **Gender:** 56% men, 44% women  **Location:** Mozambique |
| Interventions | Participants were randomly assigned to:   1. Experimental arm (*n* = 66)   **Duration:** 1 occasionally 2 individual sessions, about 60 minutes  **Treatment protocol:** testimony writing: references to ’testimony method’ but no mention of protocol  **Practitioners:** first study author interpreted into Chi-Gorongese by native speakers (same gender as participant)   1. Comparator arm (*n* = 71)   **Duration:** none  **Treatment protocol:** no intervention  **Practitioners:** none |
| Outcomes | **Time points for assessment:** pretreatment and at 11-month follow-up  **Assessment language:** all via structured interview, as participants illiterate, interpreted  into Chi-Gorongese  **Outcomes (not specified as primary or secondary):** Post-traumatic stress symptoms: self-Inventory for PTSD; only Western data available on performance of scale, Psychiatric symptoms: Self-Report Questionnaire, validated in non-Western populations, Nightmares: Nocturnal Intrusions after Traumatic Experiences Questionnaire; only Western data available on performance of scale |
| Baseline Characteristics | 58% intervention group and 55% comparator arm group tortured; many other relevant events of organised violence on HTQ, validated in non-Western populations. Mean 15 years in war zone. Mean 4 living children and 3 dead |
| Adherence and Completion | 6 dropouts |
| Notes | **Date of study:** not given  **Funding source:** part by Associação Esperança Para Todos, Mozambique  **Declarations of interest among primary researchers:** none  Third arm (not included here) of non-case participants |
| ***Risk of bias*** | |
| **Bias** | **Author’s judgement and support for judgement** |
| Random sequence generation (selection bias) | **Unclear risk**. Participants given consecutive numbers, divided according to caseness, then allocated to treatment or comparator arm according to odd or even number |
| Allocation concealment (selection bias) | **High risk**. None |
| Blinding of participants and personnel (performance bias) | **High risk.** Not possible to render participants nor practitioners blind to allocation. Expectations of benefit not accessed |
| Blinding of outcome assessment (detection bias) | **Unclear risk.** Unclear who conducted assessments – all by interview |
| Incomplete outcome data (attrition bias) | **Unclear risk**. Several dropouts (death, moving away) |
| Selective reporting (reporting bias) | **Unclear risk.** All outcomes reported in trial methods: no protocol available |
| Therapist allegiance | **Unclear risk.** No information provided |
| Treatment fidelity | **Unclear risk**. No information provided |
| Therapist qualifications | **Unclear risk.** No information provided |
| Other bias | **Unclear risk.** Real-time translation of assessment measures, so not standardised |

**Neuner et al 2010 [7]**

| Methods | Study design: randomised controlled trial |
| --- | --- |
| Participants | 32 adult outpatients at German refugee clinic from Turkey, Balkans, Africa; seeking asylum  **Diagnosis:** none  **Age:** mean age 31.3 years (SD 7.7)  **Gender:** 69% men, 31% women  **Location:** Germany |
| Interventions | Participants were randomly assigned to:   1. Experimental arm (*n* = 16)   **Duration:** median 9 individual sessions of 2 hours each  **Treatment protocol:** NET, manualised  **Practitioners:** trained, experienced therapist, observed by expert, with interpreters   1. Comparator arm (*n* = 16)   **Duration:** variable  **Treatment protocol:** treatment as usual  **Practitioners:** not given |
| Outcomes | **Time points for assessment:** pretreatment and at 6-month follow-up  **Assessment language:** used trained interpreters  **Primary outcome:** Posttraumatic Diagnostic Scale, clinician-administered, for symptom frequency  **Secondary outcome:** Diagnosis of PTSD using DSM-IV in combination with Posttraumatic Diagnostic Scale, Pain symptoms total using the Composite International Diagnostic Interview, HSCL-25 Depression Scale  No comments on use of measures in non-Western populations |
| Baseline Characteristics | All survivors of organised violence: 28 had been tortured. Mean 55 months in exile; 5 in each group still applying for asylum, and others refused asylum but granted temporary leave to remain because of mental health. Mean 7 years of education |
| Adherence and Completion | 2 dropped out of NET, none from comparator arm |
| Notes | **Date of study:** not given  **Funding source:** Deutsche Forschungsgemeinschaft  **Declarations of interest among primary researchers:** no declaration |
| ***Risk of bias*** | |
| **Bias** | **Author’s judgement and support for judgement** |
| Random sequence generation (selection bias) | **Unclear risk**. “Participants were randomized into the two groups using a block permutation procedure with blocks of four patients” |
| Allocation concealment (selection bias) | **Unclear risk**. No information provided |
| Blinding of participants and personnel (performance bias) | **High risk.** Not possible to render participants nor practitioners blind to allocation. Expectations of benefit not accessed |
| Blinding of outcome assessment (detection bias) | **Unclear risk.** Tried to keep interviewers for post-treatment assessment blind to condition, but some unblinded unwittingly by participants |
| Incomplete outcome data (attrition bias) | **Low risk**. Used mixed-effect models for missing data on the 2 who dropped out of NET |
| Selective reporting (reporting bias) | **Unclear risk.** All outcomes reported in trial methods: no protocol available |
| Therapist allegiance | **High risk.** Allegiance to NET (intervention arm) |
| Treatment fidelity | **Low risk**. Manual |
| Therapist qualifications | **Low risk.** Therapists qualified |
| Other bias | **Unclear risk.** Real-time translation of assessment measures, so not standardised. Asylum status of most participants not yet determined; possible incentive to underreport improvement |

**Paunovic & Öst 2001 [8]**

| Methods | Study design: randomised controlled trial |
| --- | --- |
| Participants | 16 outpatients referred from psychiatric units and torture survivor treatment centre; refugees but no information about countries of origin  **Diagnosis:** PTSD  **Method of diagnosis:** clinician-administered scale IV PTSD scale  **Age:** mean 37.9 years (SD 7.6)  **Gender:** 85% men, 15% women  **Location:** Sweden |
| Interventions | Participants were randomly assigned to:   1. Experimental arm (*n* = 7)   **Duration:** 16 to 20 weekly individual sessions of 1 to 2 hours plus homework  **Treatment protocol:** CBT, including exposure  **Practitioners:** doctoral student in clinical psychology, supervised by qualified and experienced clinical psychologist   1. Comparator arm (*n* = 9)   **Duration:** 16 to 20 weekly individual sessions of 1 to 2 hours plus homework  **Treatment protocol:** exposure  **Practitioners:** doctoral student in clinical psychology, supervised by qualified and experienced clinical psychologist  All therapy in Swedish, in which participants were sufficiently fluent |
| Outcomes | **Time points for assessment:** pretreatment, post treatment and at 6-month follow-up  **Assessment language:** all in Swedish, in which participants were sufficiently fluent  **Primary outcome:** clinician-administered scale IV for total PTSD severity  **Secondary outcome:** clinician-administered scale IV for global PTSD severity, Hamilton Anxiety Scale, Hamilton Depression Scale, PTSD Symptom Scale Self-Report of PTSD Symptoms, Impact of Events Scale of PTSD Symptoms, Beck Anxiety Inventory, State-Trait Anxiety Inventory, Beck Depression Inventory, World Assumptions Scale for Cognitive Schemata, Quality of Life Inventory for satisfaction weighted by importance. No reference to validation in non-Western populations |
| Baseline Characteristics | 6 torture survivors and others had combat experience or witnessed traumatic events  12 married/steady relationship, 3 single, 1 divorced; 3 full-time work, 7 unemployed, 6  long-term sick leave; 10 up to high school education and 6 some university education  75% given steady dose of psychoactive drugs |
| Adherence and Completion | 4 early dropouts/exclusion: 1 exposure and 2 CBT non-attendance; 1 CBT hostility to therapist |
| Notes | **Date of study:** not given  **Funding source:** none stated  **Declarations of interest among primary researchers:** no declaration |
| ***Risk of bias*** | |
| **Bias** | **Author’s judgement and support for judgement** |
| Random sequence generation (selection bias) | **Unclear risk**. The patients were randomly assigned to CBT or exposure, with the provision that no more than two consecutive patients would be randomised to the same condition |
| Allocation concealment (selection bias) | **Unclear risk**. No information provided |
| Blinding of participants and personnel (performance bias) | **High risk.** Not possible to render participants nor practitioners blind to allocation. Expectations of benefit not accessed |
| Blinding of outcome assessment (detection bias) | **Unclear risk.** All by self-report; no third party assessment |
| Incomplete outcome data (attrition bias) | **High risk.** 4 dropouts excluded from analyses |
| Selective reporting (reporting bias) | **Unclear risk.** All outcomes reported in trial methods: no protocol available |
| Therapist allegiance | **Unclear risk.** No information provided |
| Treatment fidelity | **Unclear risk**. No information provided |
| Therapist qualifications | **Unclear risk.** Therapist in training |
| Other bias | **Unclear risk.** None |

**Pokhariyal et al 2013 [9]**

| Methods | Study design: randomised controlled trial |
| --- | --- |
| Participants | 96 survivors of torture: 43 Kenyan torture survivors recruited from People Against Torture  or released Kenyan political prisoners and 53 refugees in Kenya under United Nations High Commissioner for Refugees programme  **Diagnosis:** none  **Age:** Kenyans mean 36.9 years (SD 11.5); refugees mean 26.7 years (SD 6.5)  **Gender:** Kenyans 81% men, 19% women; refugees 51% men, 49% women  **Location:** Kenya |
| Interventions | Participants were randomly assigned to:   1. Experimental arm (*n* = 31)   **Duration:** mean 5 individual sessions of multi-sensory trauma processing + 5 participants had 1 to 3 sessions of conventional treatment  **Treatment protocol:** Multi-sensory trauma processing +/- conventional treatment  **Practitioners:** members of research team, all experienced and qualified in counselling psychology   1. Comparator arm (*n* = 38)   **Duration:** mean 9 individual sessions  **Treatment protocol:** conventional treatment = “eclectic methods of psychotherapy”: an assortment of therapeutic techniques with varied or no evidence of efficacy  **Practitioners:** members of research team, all experienced and qualified in counselling psychology  Interpreted into Kiswahili or Kikuyu for Kenyan participants when necessary |
| Outcomes | **Time points for assessment:** pretreatment, post treatment  **Assessment language:** Kiswahili or Kikuyu for Kenyan participants; some used interpreters  **Primary outcome:** Stress State Inventory (items on PTSD symptoms)  **Secondary outcome:** none  Stress State Inventory developed for US veterans: no comment on cross-cultural validity |
| Baseline Characteristics | Partial data only. Kenyans (N = 26): 18 educated up to secondary level, 7 beyond; 17 married, 6 single, 3 divorced/widowed; 16 Christian, 5 Muslim, 5 other. Refugees (N = 30): educated up to secondary level, 10 beyond; 30 married, 17 single, 2 divorced/widowed; 20 Christian, 26 Muslim, 3 other |
| Adherence and Completion | 27 “excluded for various reasons”: 35 Kenyans and 34 refugees completed |
| Notes | **Date of study**: not given  **Funding source:** United States Agency for International Development, United States International University Africa  **Declarations of interest among primary researchers:** none  Kenyan and refugee participants had somewhat different baseline scores and received different doses of treatment, but we combined them for analysis. Data were provided individually per subject in tables, so means and standard deviations were calculated |
| ***Risk of bias*** | |
| **Bias** | **Author’s judgement and support for judgement** |
| Random sequence generation (selection bias) | **Unclear risk**. Names of recruits converted to numbers and then “randomly assigned” Kenyans and refugees separately. No further detail |
| Allocation concealment (selection bias) | **Unclear risk**. No information provided |
| Blinding of participants and personnel (performance bias) | **High risk.** Not possible to render participants nor practitioners blind to allocation. Expectations of benefit not accessed |
| Blinding of outcome assessment (detection bias) | **Unclear risk.** Self-report measure but described as “administered,” so unclear |
| Incomplete outcome data (attrition bias) | **High risk.** Only completers analysed |
| Selective reporting (reporting bias) | **Unclear risk.** Single outcome measure in trial reported; no protocol available |
| Therapist allegiance | **Unclear risk.** No information provided |
| Treatment fidelity | **Unclear risk**. No information provided |
| Therapist qualifications | **Low risk.** Qualified therapists |
| Other bias | **Unclear risk.**  Real-time translation of assessment measures, so not standardised |

**Puvimanasinghe and Price 2016 [10]**

| Methods | **Study design**: randomised controlled trial |
| --- | --- |
| Participants | 26 primary (69%) and secondary survivors of torture (30%)  **Diagnosis**: none specified – distress measured instead  **Method of diagnosis:** not applicable  **Age:** mean 40.5 years  **Gender**: 38.5% female  **Location**: Sri Lanka |
| Interventions | Participants were randomly assigned to:   1. Experimental arm (*n* = 13)   **Duration**: 5 sessions (60-90 minutes each)  **Treatment protocol** testimony therapy plus ceremony  **Practitioners**: counsellors trained in testimony therapy plus ceremony from a German clinician, with biweekly supervision from psychologist in Cambodia.   1. Comparator arm: (*n* = 13)   **Duration**: waitlist control: not described  **Treatment protocol**: participants were informed that they would be contacted again and offered testimony therapy at a later date  **Practitioners**: no information available |
| Outcomes | **Time points for assessment**: baseline and 2-3 months later  **Assessment language**: Sinhalese  **Primary outcome**: trauma-related distress (Sri Lanka Index of Psychosocial Status), social participation (Participation scale)  **Secondary outcome**: emotional well-being (World Health Organisation Five Well-being Index) to measure depression |
| Baseline Characteristics | In both groups, 77% of participants experienced torture, 15.4% experienced ill-treatment and 7.7% experienced psychological abuse. Comparison of mean pre-test scores for the two groups found no significant differences for all outcomes, and no age or gender differences. |
| Adherence and Completion | No participants were lost to follow-up |
| Notes |  |
| ***Risk of bias*** | |
| **Bias** | **Author’s judgement and support for judgement** |
| Random sequence generation (selection bias) | **Unclear risk**. Authors state random assignment but provide no detail on methods |
| Allocation concealment (selection bias) | **High risk**. Assignment to condition was decided by using the characteristics of gender, participant status, and nature of violation, to match pairs of participants as closely as possible |
| Blinding of participants and personnel (performance bias) | **High risk**. Not possible to render participants nor practitioners blind to ­­­allocation |
| Blinding of outcome assessment (detection bias) | **Unclear risk**. Method not described |
| Incomplete outcome data (attrition bias) | **Low risk.** No loss of participants, some outcomes not completed but intention-to-treat analyses were used |
| Selective reporting (reporting bias) | **Low risk.** All measures were used and reported but no protocol reported |
| Therapist allegiance | **Unclear risk.** Counsellors prior experience, training and qualifications not reported |
| Treatment fidelity | **Unclear risk**. No information provided |
| Therapist qualifications | **Unclear risk**. Counsellors’ experience and qualifications not reported |
| Other bias | **Unclear risk.** |

**Schauer et al 2006 [11]**

| Methods | **Study design**: randomised controlled trial |
| --- | --- |
| Participants | 32 outpatients in refugee trauma clinic; mostly Kurdish; asylum seekers awaiting determination  of asylum claim  **Diagnosis:** PTSD  **Method of diagnosis:** DSM-IV  **Age:** mean age 31.3 years (SD 7.7)  **Gender:** 69% men, 31% women  **Location:** Germany |
| Interventions | Participants were randomly assigned to:  1. Experimental arm  **Duration:** no information  **Treatment protocol:** NET, manualised  **Practitioners:** no information  2. Comparator arm  **Duration:** no information  **Treatment protocol:** treatment as usual (various psychotherapies ± pharmacotherapy)  **Practitioners:** no information  Interpreters used for all |
| Outcomes | **Time points for assessment:** pretreatment, post treatment and at 6-month follow-up  **Assessment language:** various, interpreted  **Primary outcome:** Posttraumatic Diagnostic Scale  **Secondary outcome:** None |
| Baseline Characteristics | More than half described torture experiences with average of 4 to 5 traumatic events in prison or detention. Mean 7 years of education, median 2 children. Some taking medication |
| Adherence and Completion | All completed |
| Notes | **Date of study:** not given  **Funding source:** none  **Declarations of interest among primary researchers:** no declaration  Data provided by first study author |
| ***Risk of bias*** | |
| **Bias** | **Author’s judgement and support for judgement** |
| Random sequence generation (selection bias) | **Unclear risk**. No information provided |
| Allocation concealment (selection bias) | **Unclear risk**. No information provided |
| Blinding of participants and personnel (performance bias) | **High risk**. Not possible to render participants nor practitioners blind to ­­­allocation |
| Blinding of outcome assessment (detection bias) | **High risk**. Self-report |
| Incomplete outcome data (attrition bias) | **Low risk.** No attrition |
| Selective reporting (reporting bias) | **Unclear risk.** Single outcome in trial reported: no protocol available |
| Therapist allegiance | **High risk.** Allegiance to NET (intervention arm) |
| Treatment fidelity | **Low risk**. Manual |
| Therapist qualifications | **Unclear risk**. No information provided |
| Other bias | **Unclear risk.** Asylum status of participants undecided; may act as incentive to underreport improvement |

**ter Heide et al 2011 [12]**

| Methods | **Study design**: randomised controlled trial; pilot study for larger trial |
| --- | --- |
| Participants | 20 outpatients of trauma clinic; asylum seekers or refugees from Europe, Middle East,  Africa  **Diagnosis:** PTSD  **Method of diagnosis:** modified diagnostic criteria for PTSD  **Age:** mean age 41.5 years (SD 8.8)  **Gender:** 60% men, 40% women  **Location:** Netherlands |
| Interventions | Participants were randomly assigned to:   1. Experimental arm (*n* = 5)   **Duration:** 11 individual sessions weekly or biweekly  **Treatment protocol:** Eye movement desensitisation and reprocessing: “A therapist manual was designed containing information on study design and guidelines on therapy content”  **Practitioners:** trained and 1 session evaluated by supervisor   1. Comparator arm (*n* = 5)   **Duration:** 11 individual sessions, weekly or biweekly  **Treatment protocol:** stabilisation (present-centred therapy; no exposure)  **Practitioners:** various disciplines, supervised monthly  Interpreters used for 3 in each arm; treatments evaluated using fidelity scales |
| Outcomes | **Time points for assessment:** pretreatment, post treatment and at 3-month follow-up  **Assessment language**: Dutch trained by blind assessors with interpreters as necessary; “Linguistic difficulties resulted in eight participants needing an interpreter during assessments and three needing extensive help with filling in the questionnaires.”  **Primary outcome**: PTSD symptoms, Structured Clinical Interview (clinician-administered)  **Secondary outcome**: HTQ for PTSD symptoms, clinician administered, HSCL-25 for Anxiety, self-report, HSCL-25 for Depression, self-report  WHOQOL-BREF for Quality of Life, self-report. “HTQ, HSCL-25, and WHOQOL-BREF are self-report questionnaires that are widely used with this population and are available in many different languages. All three have good psychometric properties” |
| Baseline Characteristics | 14 reported torture, 17 residency status granted; mean 10 years in Netherlands, 11 married; 8 primary school education or less; 6 employed |
| Adherence and Completion | 10 (5 in each condition) dropped out. 3 satisfied with symptom reduction, but others disliked methods, symptoms worsened or missing sessions |
| Notes | **Date of study:** 2007  **Funding source:** part ZonMW, Netherlands  **Declarations of interest among primary researchers:** none |
| ***Risk of bias*** | |
| **Bias** | **Author’s judgement and support for judgement** |
| Random sequence generation (selection bias) | **Low risk**. “Blocking was applied, with blocks of the latest two patients who had satisfied inclusion criteria. Participants were assigned to their experimental group using simple  randomisation through flipping a coin: the outcome (Eye movement desensitisation and reprocessing for heads, stabilisation for tails) was assigned to the patient lowest in the alphabet. An independent research associate performed randomisation” |
| Allocation concealment (selection bias) | **Unclear risk**. No information provided |
| Blinding of participants and personnel (performance bias) | **High risk**. Not possible to render participants nor practitioners blind to ­­­allocation |
| Blinding of outcome assessment (detection bias) | **Unclear risk**. 33/44 assessments maintained blind (using Structured Clinical Interview) for primary outcome; secondary outcomes by self-report |
| Incomplete outcome data (attrition bias) | **High risk.** Analysis of completers only |
| Selective reporting (reporting bias) | **Unclear risk.** All outcomes in trial reported: no protocol available |
| Therapist allegiance | **Unclear risk.** Allegiance possibly to eye movement desensitisation and reprocessing |
| Treatment fidelity | **Low risk**. Manual produced for trial |
| Therapist qualifications | **Low risk**. Trained therapists |
| Other bias | **Unclear risk.** Real-time translation of assessment measures, so not standardised |

**Wang et al 2016 [13]**

| Methods | **Study design**: pilot randomised controlled trial |
| --- | --- |
| Participants | 34 torture and war victims with chronic pain and comorbid mental health diagnosis  **Diagnosis**: chronic pain, PTSD, depression or anxiety  **Method of diagnosis:** Wong-Baker FACES Pain Rating Scale and Short-form McGill Pain Questionnaire to assess chronic pain**,** HTQ for PTSD, HSCL-25 for depression and anxiety symptoms  **Age:** mean 47.7 years  **Gender**: 45% female  **Location**: Kosovo |
| Interventions | Participants were randomly assigned to:   1. Experimental arm (*n* = 17)   **Duration**: 10 weekly individual sessions (90m duration) and 10 weekly group sessions (60-90m duration) over 3 months  **Treatment Protocol**: CBT with adapted prolonged exposure and breathing exercises using biofeedback, group physiotherapy and daily multivitamin  **Practitioners**: 3 therapists (1 doctor and 2 psychologists) and 3 physiotherapists   1. Comparator arm (n = 17)   **Duration**: 3 months  **Treatment protocol**: waitlist control: daily multivitamin  **Practitioners**: same as above |
| Outcomes | **Time points for assessment**: baseline, 3 months, 6 months  **Assessment language**: Albanian  **Primary outcome:** PTSD symptoms (HTQ), anxiety and depression (HSCL-25)  **Secondary outcome**: chronic pain (FACES and Short-form McGill Pain Questionnaire), functioning and quality of life (World Health Organization Disability Assessment Schedule) |
| Baseline Characteristics | Participants in the intervention arm had worse baseline symptoms of chronic pain and disability than participants in comparator arm, while participants with chronic pain or depression were equally distributed in both groups |
| Adherence and Completion | 2 participants did not begin the treatment, and 1 dropped out before the second session. Outcome data for 6 participants had systematically missing data at month 6. In total, 13 participants in the intervention group and 15 in the waiting list group were included in an intent-to-treat analysis. Attendance rates were 76% and 55% for individual sessions and group respectively, with no significance difference in attendance between both groups |
| ***Risk of bias*** | |
| **Bias** | **Author’s judgement and support for judgement** |
| Random sequence generation (selection bias) | **Low risk**. A block randomisation procedure using a computerised random number generator created was used by staff member not involved in the trial |
| Allocation concealment (selection bias) | **Low risk.** Each participant was given a unique number according to above method |
| Blinding of participants and personnel (performance bias) | **High risk.** Not possible render both participants and therapists blind to group allocation |
| Blinding of outcome assessment (detection bias) | **Low risk**. “Participants and therapists were blinded to allocation and therapists were blinded to outcomes during assessments” |
| Incomplete outcome data (attrition bias) | **Low risk.** Intent to treat analysis as 3 participants dropped out and 6 participants had systematically missing data |
| Selective reporting (reporting bias) | **Unclear risk**. All measures were used and reported but more outcomes reported than described) and no protocol reported |
| Therapist allegiance | **Unclear risk.** Inadequate information of qualifications and experience |
| Treatment fidelity | **Unclear risk**. 15% of CBT treatment sessions were randomly selected, recorded and reviewed. “But no details given by authors. A physiotherapy manual was given to physiotherapists for the group, but sessions were not monitored closely |
| Therapist qualifications | **Unclear risk**. There was variable pre-trial experience |
| Other bias | **Unclear risk.** |

**Weiss et al 2015 [14]**

| Methods | **Study design**: two-site, two-armed randomised controlled trial |
| --- | --- |
| Participants | 342 torture survivors  **Diagnosis**: PTSD  **Method of diagnosis:** HTQ to assess trauma symptoms, HSCL-25 to assess depression and anxiety symptoms  **Age:** mean 41.9 years  **Gender**: 33% female  **Location**: Southern Iraq |
| Interventions | Participants were randomly assigned to:   1. Experimental arm 1 (*n* = 99)   **Duration**: 8-12 weekly sessions  **Treatment Protocol**: common elements approach  **Practitioners**: 12 non-specialised CMHWs working in South Iraq   1. Comparator arm for experimental arm 1 (*n* = 50)   **Duration**: 12 weeks  **Treatment protocol**: waitlist control: monthly telephone calls from CMHWs who enrolled them into the study to assess safety  **Practitioners**: same as above   1. Experimental arm 2 (*n* = 129)   **Duration**: 12 weekly sessions  **Treatment protocol** cognitive processing therapy  **Practitioners**: 17 non-specialised CMHWs working in South Iraq   1. Comparator arm for experimental arm 2 (*n* = 64)   **Duration**: 12 weeks  **Treatment protocol**: waitlist control: monthly telephone calls from CMHWs who enrolled them into the study to assess safety.  **Practitioners**: same as above |
| Outcomes | **Time points for assessment**: baseline, post-intervention. Mean time from end of treatment to follow-up interview is 3.5 months for common elements approach and 4.5 months for cognitive processing therapy  **Assessment language**: Arabic  **Primary outcome:** PTSD symptoms (HTQ)  **Secondary outcome**: functional impairment, assessed by a locally developed scale described elsewhere, anxiety and depression assessed by HSCL-25 |
| Baseline Characteristics | For common elements approach arm, controls tended to be older (mean age 45.2 years versus 41.6 years), less likely to be single (4% versus 13%) and less likely to have a disability (2% versus 13%) than intervention group. The researchers did not identify any differences in trauma, anxiety, depression, dysfunction and other demographic variables. For cognitive processing therapy arm, there were no differences in characteristics between intervention and comparator clients |
| Adherence and Completion | Of the 99 participants enrolled in the common elements approach arm, 97 (98%) completed therapy and all 97 were reassessed at follow-up. Of the 50 comparator participants, all completed a follow-up interview but interview forms for 2 participants were lost. Of the 129 participants enrolled in the cognitive processing therapy arm, 107 (82.9%) completed therapy, and all but 1 completed follow-up. 18 intervention drop outs also completed follow-up interviews. Of the 64 comparator participants, all completed follow-up |
| Notes | Common elements approach and cognitive processing therapy manual was translated and adapted for the Southern Iraq context |
| ***Risk of bias*** | |
| **Bias** | **Author’s judgement and support for judgement** |
| Random sequence generation (selection bias) | **Low risk.** Separate randomisation lists for each mental health worker was produced using a random number generator in Microsoft Excel |
| Allocation concealment (selection bias) | **Unclear risk**. Allocation was stapled to the back of consent form |
| Blinding of participants and personnel (performance bias) | **High risk.** Not possible to render participants nor practitioners blind to allocation – “the supervisors and study participants were not blind to the treatment condition” |
| Blinding of outcome assessment (detection bias) | **Low risk**. Baseline assessments were conducted by CMHWs who were blind to assignment of study and different CMHW assessors were used from baseline to follow-up |
| Incomplete outcome data (attrition bias) | **Unclear risk.** Overall high adherence to therapy but greater attrition in intervention arms relative to comparator arms |
| Selective reporting (reporting bias) | **Low risk.** All measures were used and reported, validity and reliability tests reported but no protocol reported |
| Therapist allegiance | **Unclear risk**. Although all CMHWs were non-specialised and received the same training, no further information is provided |
| Treatment fidelity | **Low risk.** “Fidelity was tracked by CMHW self-report of elements delivered, supervisor review of notes and CMHW reports, and finally by trainer review” |
| Therapist qualifications | **Unclear risk.** CMHW primarily medics or nurses who were trained in counselling methods but no specific information provided about CMHWs training and qualifications |
| Other bias | **Unclear risk.** Variation across treatment that participants received according to therapist’s judgement of need |

**Yeomans et al 2010 [15]**

| Methods | **Study design**: randomised controlled trial |
| --- | --- |
| Participants | 124 refugees in Internally Displaced Persons camps, referred by church elders  **Diagnosis:** none  **Age:** mean age 38.6 years (SD 12.8)  **Gender:** 56% men, 44% women  **Location:** Burundi |
| Interventions | Participants were randomly assigned to:   1. Experimental arm (*n* = 75)   **Duration:** 3-day group workshop plus 1 day 1 month later  **Treatment protocol:** 2 arms combined: trauma healing and reconciliation with PTSD psychoeducation, and trauma healing and reconciliation. Both described as standardised and drew on several manuals  **Practitioners:** Burundian facilitators, experienced in workshops and briefly trained for this trial   1. Comparator arm (*n* = 38)   **Duration:** none  **Treatment protocol:** waiting list control  **Therapist:** none  Therapy in participants’ own language |
| Outcomes | **Time points for assessment:** pretreatment, post treatment  **Assessment language**: all translated in Kirundi, back-translated into English, compared, adjusted and subjected to expert linguistic scrutiny. Administered orally, as most participants  illiterate  **Primary outcome:** HTQ Part IV (HTQa) for PTSD symptoms, self-report orally. They refer to previous use and Cronbach’s alpha in similar population  **Secondary outcomes**: HTQ additional items for emotional state related to trauma (HTQb) self-report orally, HSCL-25 for Anxiety and Depression; 10 additional items in HSCL format for somatic distress, self-report orally. They refer to cultural sensitivity and previous use and Cronbach’s alpha in similar population |
| Baseline Characteristics | Almost all participants had been directly victimized by violence during or since the conflict in 1993” 95% < 7 years of education; 52.4% Hutu, 47.6% Tutsi |
| Adherence and Completion | 3 dropouts and 4 further losses to assessment post treatment in groups analysed |
| Notes | **Date of study:** 2007  **Funding source:** none  **Declarations of interest among primary researchers:** no declaration  Data analysed from trauma healing with and without psychoeducation vs waiting list control |
| ***Risk of bias*** | |
| **Bias** | **Author’s judgement and support for judgement** |
| Random sequence generation (selection bias) | **Unclear risk.** “Participants were blocked according to ethnicity and gender and randomly assigned to condition” |
| Allocation concealment (selection bias) | **Unclear risk**. No information provided |
| Blinding of participants and personnel (performance bias) | **High risk.** Not possible to render participants nor practitioners blind to allocation. Expectations of benefit not assessed |
| Blinding of outcome assessment (detection bias) | **Low risk**. Assessment by self-report: interviewers blind to allocation |
| Incomplete outcome data (attrition bias) | **High risk.** Only completers analysed |
| Selective reporting (reporting bias) | **Unclear risk.** All outcomes in trial reported: no protocol available |
| Therapist allegiance | **Unclear risk**. No information provided |
| Treatment fidelity | **Unclear risk.** No information provided |
| Therapist qualifications | **Low risk.** Therapists qualified |
| Other bias | **Unclear risk.** Real-time translation of assessment measures, so not standardised |

**References**

1. Bass J, Murray SM, Mohammed TA, Bunn M, Gorman W, Ahmed AM, et al. A randomized controlled trial of a trauma-informed support, skills, and psychoeducation intervention for survivors of torture and related trauma in Kurdistan, Northern Iraq. Glob Health Sci Pract. 2016 Sep 28;4(3):452-66.
2. Bichescu D, Neuner F, Schauer M, Elbert T. Narrative exposure therapy for political imprisonment-related chronic posttraumatic stress disorder and depression. Behav Res Ther. 2007 Sep 1;45(9):2212-20.
3. Bolton P, Lee C, Haroz EE, Murray L, Dorsey S, Robinson C, et al. A transdiagnostic community-based mental health treatment for comorbid disorders: development and outcomes of a randomized controlled trial among Burmese refugees in Thailand. PLOS Med. 2014 Nov 11;11(11):e1001757.
4. Esala JJ, Taing S. Testimony therapy with ritual: a pilot randomized controlled trial. J Trauma Stress. 2017 Feb;30(1):94-8.
5. Hensel-Dittmann D, Schauer M, Ruf M, Catani C, Odenwald M, Elbert T, et al. Treatment of traumatized victims of war and torture: a randomized controlled comparison of narrative exposure therapy and stress inoculation training. Psychother Psychosom. 2011;80(6):345-52.
6. Igreja V, Kleijn WC, Schreuder BJ, Van Dijk JA, Verschuur M. Testimony method to ameliorate post-traumatic stress symptoms: Community-based intervention study with Mozambican civil war survivors. Br J Psychiatry. 2004 Mar;184(3):251-7.
7. Neuner F, Kurreck S, Ruf M, Odenwald M, Elbert T, Schauer M. Can asylum-seekers with posttraumatic stress disorder be successfully treated? A randomized controlled pilot study. Cogn Behav Ther. 2010 Jan 1;39(2):81-91.
8. Paunovic N, Öst LG. Cognitive-behavior therapy vs exposure therapy in the treatment of PTSD in refugees. Behav Res Ther. 2001 Oct 1;39(10):1183-97.
9. Pokhariyal GP, Rono R, Munywoki S. Analysis of treatment methods for victims of torture in Kenya and East Africa region. Traumatology. 2013 Jun;19(2):107-17.
10. Puvimanasinghe TS, Price IR. Healing through giving testimony: An empirical study with Sri Lankan torture survivors. Transcult Psychiatry. 2016 Oct;53(5):531-50.
11. Schauer M, Elbert T, Gotthardt S, Rockstroh B, Odenwald M, Neuner F. Imaginary reliving in psychotherapy modifies mind and brain. Verhaltenstherapie. 2006 Jan 1;16(2):96-103.
12. ter Heide FJ, Mooren T, Kleijn W, de Jongh A, Kleber R. EMDR versus stabilisation in traumatised asylum seekers and refugees: Results of a pilot study. Eur J Psychotraumatol. 2011 Jan 1;2(1):5881.
13. Wang SJ, Bytyçi A, Izeti S, Kallaba M, Rushiti F, Montgomery E, et al. A novel bio-psycho-social approach for rehabilitation of traumatized victims of torture and war in the post-conflict context: a pilot randomized controlled trial in Kosovo. Confl Health. 2016 Dec;10(1):34.
14. Weiss WM, Murray LK, Zangana GA, Mahmooth Z, Kaysen D, Dorsey S, et al. Community-based mental health treatments for survivors of torture and militant attacks in Southern Iraq: a randomized control trial. BMC Psychiatry. 2015 Dec;15(1):249.
15. Yeomans PD, Forman EM, Herbert JD, Yuen E. A randomized trial of a reconciliation workshop with and without PTSD psychoeducation in Burundian sample. J Trauma Stress. 2010 Jun;23(3):305-12.
